# Acute Kidney Injury and Renal Replacement Therapy in COVID-19 Versus Other Respiratory Viruses: A Systematic Review and Meta-Analysis

**DOI:** 10.1177/20543581211052185

**Published:** 2021-10-30

**Authors:** A. Cau, M. P. Cheng, Terry Lee, A. Levin, T. C. Lee, D. C. Vinh, F. Lamontagne, J. Singer, K. R. Walley, S. Murthy, D. Patrick, O. Rewa, B. Winston, J. Marshall, J. Boyd, JA Russell

**Affiliations:** 1 The University of British Columbia, Vancouver, BC, Canada; 2 Department of Medicine, McGill University, Montreal, QC, Canada; 3 Centre for Health Evaluation & Outcomes Science, The University of British Columbia, Vancouver, BC, Canada; 4 Division of Nephrology, St. Paul’s Hospital, Vancouver, BC, Canada; 5 University of Sherbrooke, QC, Canada; 6 Centre for Heart Lung Innovation, St. Paul’s Hospital and The University of British Columbia, Vancouver, BC, Canada; 7 BC Children’s Hospital, The University of British Columbia, Vancouver, BC, Canada; 8 British Columbia Centre for Disease Control and The University of British Columbia, Vancouver, BC, Canada; 9 University of Alberta, Edmonton, AB, Canada; 10 University of Calgary, Calgary, AB, Canada; 11 St. Michael’s Hospital, University of Toronto, Toronto, ON, Canada

**Keywords:** acute kidney injury, renal replacement therapy, coronavirus disease-2019, COVID-19, angiotensin converting enzyme 2

## Abstract

**Background:**

Acute kidney injury (AKI) is a potentially fatal complication of Coronavirus Disease-2019 (COVID-19). Binding of the Severe Acute Respiratory Syndrome Coronavirus 2 (SARS-CoV-2), the virus responsible for COVID-19, to its viral receptor, angiotensin converting enzyme 2 (ACE2), results in viral entry and may cause AKI.

**Objectives:**

We performed a systematic review and meta-analysis of the frequencies of AKI and renal replacement therapy (RRT) in critically ill COVID-19 patients and compared those frequencies with patients who were infected by respiratory viruses that bind or downregulate ACE2 (ACE2-associated viruses) and viruses that do not bind nor downregulate ACE2 (non-ACE2-associated viruses).

**Design:**

Systematic review and meta-analysis.

**Setting:**

Observational studies on COVID-19 and other respiratory viral infections reporting AKI and RRT were included. The exclusion criteria were non-English articles, non-peer-reviewed articles, review articles, studies that included patients under the age of 18, studies including fewer than 10 patients, and studies not reporting AKI and RRT rates.

**Patients:**

Adult COVID-19, Severe Acute Respiratory Syndrome (SARS), Middle East Respiratory Syndrome (MERS), and influenza patients.

**Measurements:**

We extracted the following data from the included studies: author, year, study location, age, sex, race, diabetes mellitus, hypertension, chronic kidney disease, shock, vasopressor use, mortality, intensive care unit (ICU) admission, ICU mortality, AKI, and RRT.

**Methods:**

We systematically searched PubMed and EMBASE for articles reporting AKI or RRT. AKI was defined by authors of included studies. Critical illness was defined by ICU admission. We performed a random effects meta-analysis to calculate pooled estimates for the AKI and RRT rate within each virus group using a random intercept logistic regression model.

**Results:**

Of 23 655 hospitalized, critically ill COVID-19 patients, AKI frequencies were not significantly different between COVID-19 patients (51%, 95% confidence interval [CI]: 44%-57%) and critically ill patients infected with ACE2-associated (56%, 95% CI: 37%-74%, *P* = .610) or non-ACE2-associated viruses (63%, 95% CI: 43%-79%, *P* = .255). Pooled RRT rates were also not significantly different between critically ill, hospitalized patients with COVID-19 (20%, 95% CI: 16%-24%) and ACE2-associated viruses (18%, 95% CI: 8%-33%, *P* = .747). RRT rates for both COVID-19 and ACE2-associated viruses were significantly different (*P* < .001 for both) from non-ACE2-associated viruses (49%, 95% CI: 44%-54%). After adjusting for shock or vasopressor use, AKI and RRT rates were not significantly different between groups.

**Limitations:**

Limitations of this study include the heterogeneity of definitions of AKI that were used across different virus studies. We could not match severity of infection or do propensity matching across studies. Most of the included studies were conducted in retrospective fashion. Last, we did not include non-English publications.

**Conclusions:**

Our findings suggest that viral ACE2 association does not significantly alter the rates of AKI and RRT among critically ill patients admitted to the ICU. However, the rate of RRT is lower in patients with COVID-19 or ACE2-associated viruses when compared with patients infected with non-ACE2-binding viruses, which might partly be due to the lower frequencies of shock and use of vasopressors in these two virus groups. Prospective studies are necessary to demonstrate whether modulation of the ACE2 axis with Renin-Angiotensin System inhibitors impacts the rates of AKI and whether they are beneficial or harmful in COVID-19 patients.

## Abrégé

**Mise en contexte:**

L’insuffisance rénale aiguë (IRA) est une complication potentiellement mortelle de la maladie à coronavirus-2019 (COVID-19). Obligatoire du Coronavirus 2 du Syndrome Respiratoire Aigu Sévère (SARS-CoV-2), le virus responsable du COVID-19, à son récepteur, l’enzyme de conversion de l’angiotensine 2 (ACE2), entraîne une entrée virale et peut provoquer une IRA.

**Objectifs de l’étude:**

Nous avons effectué une revue systématique et une méta-analyse des fréquences de l’IRA et de la thérapie de remplacement renal (RRT) chez les patients COVID-19 gravement malades et a comparé ces fréquences avec les patients qui ont été infectés par des voies respiratoires virus qui lient ou régulent négativement l’ACE2 (virus associés à l’ACE2) et les virus qui ne régulent pas négativement ni ne lient l’ACE2 (virus non associés à l’ACE2).

**Cadre et type d’étude:**

Revue systématique et méta-analyse. Des études d’observation sur le COVID-19 et d’autres infections virales respiratoires signalant une AKI et une RRT ont été incluses. Les critères d’exclusion étaient des articles non anglophones, des articles non évalués par des pairs, des articles de revue, des études incluant des patients moins de 18 ans, les études incluant moins de 10 patients et les études ne rapportant pas les taux d’IRA et de RRT.

**Patients:**

Adultes COVID-19, syndrome respiratoire aigu sévère (SRAS), syndrome respiratoire du Moyen-Orient (MERS) et malades de la grippe.

**Mesures:**

Nous avons extrait les données suivantes des études incluses : auteur, année, lieu de l’étude, âge, sexe, race, diabète sucré, hypertension, maladie rénale chronique, état de choc, utilisation de vasopresseurs, mortalité, admission en unité de soins intensifs (USI), Mortalité en soins intensifs, AKI et RRT.

**Méthodologie:**

Nous avons systématiquement recherché dans PubMed et EMBASE les articles rapportant AKI ou RRT. AKI a été défini par les auteurs des études incluses. La maladie grave a été définie par l’admission aux soins intensifs. Nous avons effectué une méta-analyse à effets aléatoires pour calculer estimations regroupées pour le taux d’IRA et de RRT au sein de chaque groupe de virus à l’aide d’un modèle de régression logistique d’interception aléatoire.

**Résultats:**

Sur 23 655 patients hospitalisés et gravement malades COVID-19, les fréquences AKI n’étaient pas significativement différentes entre patients COVID-19 (51 %, intervalle de confiance à 95 % [IC] : 44 %-57 %) et patients gravement malades infectés par l’ACE2 associé (56 %, IC à 95 % : 37 % à 74 %, P = 0,610) ou des virus non associés à l’ACE2 (63 %, IC à 95 % : 43 % à 79 %, *P* = 0,255). Tarifs RRT groupés n’étaient pas non plus significativement différents entre les patients hospitalisés gravement malades atteints de COVID-19 (20 %, IC à 95 % : 16 % à 24 %) et virus associés à l’ACE2 (18 %, IC à 95 % : 8 % à 33 %, P = 0,747). Taux de RRT pour les virus associés au COVID-19 et à l’ACE2 étaient significativement différents (P < 0,001 pour les deux) des virus non associés à l’ACE2 (49 %, IC à 95 % : 44 % à 54 %). Après ajustement pour le choc ou l’utilisation de vasopresseurs, les taux d’IRA et de RRT n’étaient pas significativement différents entre les groupes.

**Limites de l’étude:**

Les limites de cette étude incluent l’hétérogénéité des définitions de l’IRA qui ont été utilisées pour différents virus études. Nous n’avons pas pu faire correspondre la gravité de l’infection ou faire une correspondance de propension entre les études. La plupart des études incluses ont été menées de manière rétrospective. Enfin, nous n’avons pas inclus les publications non anglophones.

**Conclusions:**

Nos résultats suggèrent que l’association virale ACE2 ne modifie pas de manière significative les taux d’IRA et de RRT parmi les patients gravement malades admis aux soins intensifs. Cependant, le taux de RRT est plus faible chez les patients atteints de COVID-19 ou associés à l’ACE2 virus par rapport aux patients infectés par des virus ne se liant pas à l’ACE2, ce qui pourrait être dû en partie à la plus faible fréquences de choc et utilisation de vasopresseurs dans ces deux groupes de virus. Des études prospectives sont nécessaires pour démontrer si la modulation de l’axe ACE2 avec les inhibiteurs du système rénine-angiotensine a un impact sur les taux d’IRA et si ells sont bénéfiques ou nocifs chez les patients COVID-19.

## What was known before

While a small proportion of patients with Coronavirus Disease-2019 (COVID-19) were known to develop acute kidney injury or require renal replacement therapy, the true rates of acute kidney injury and renal replacement therapy in hospitalized, critically ill COVID-19 patients were unknown. Furthermore, the rates of acute kidney injury and renal replacement therapy in hospitalized patients infected with other respiratory viruses that do or do not bind or downregulate angiotensin converting enzyme 2 (ACE2) have been described in isolation but, it remains unclear whether these rates are different than those in COVID-19. The ongoing debate of whether Renin-Angiotensin System (RAS) inhibitors confer protection against or increases susceptibility to COVID-19 infection underscores the dire need to understand whether viral association with ACE2, through binding or downregulation, is correlated with the development of end-organ injury such as AKI.

## What this adds

Acute kidney injury occurs to approximately the same degree in critically ill patients, that is, patients admitted to the ICU, regardless of viral association with ACE2. The minimal impact of ACE2 association with rates of acute kidney injury restates the need for and provides a rationale for trials of RAS inhibitors for the therapeutic management of COVID-19.

## Introduction

Coronavirus-Disease 2019 (COVID-19) has resulted in over 140 000 000 cases and over 3 000 000 deaths since it was first identified in December 2019.^1-3^

Angiotensin converting enzyme 2 (ACE2) is the viral receptor for Severe Acute Respiratory Syndrome Coronavirus 2 (SARS-CoV-2).^4-6^ ACE2 cleaves angiotensin II (Ang II) into the vasodilatory, anti-inflammatory peptide angiotensin 1-7 (Ang [1-7]).^7,8^

ACE2 is also the viral receptor for SARS-CoV-1.^9^ The cellular distribution of ACE2, on the vascular endothelium, kidney, heart, and lungs, may explain, in part, the frequent occurrence of acute kidney injury (AKI), acute cardiac injury (ACI), and acute respiratory distress syndrome (ARDS) in COVID-19 patients.^10-13^ SARS-CoV-2 binding to ACE2 leads to SARS-CoV-2 entry and downregulation of ACE2 at the cell membrane, similar to SARS-CoV-1.^14^ Other respiratory viruses also downregulate ACE2 expression (eg, influenza H1N1, H5N1, H7N9).^15-17^

The main proposed mechanisms of AKI in COVID-19 include (1) renal hypoperfusion due to fever, gastrointestinal losses, cardiomyopathy, shock, and use of vasopressors, (2) direct viral injury, (3) sepsis and septic shock-associated acute tubular necrosis, (4) increased inflammation due to increased pro-inflammatory cytokines and cytokine storm, (5) rhabdomyolysis, (6) micro- and macro-vascular thrombosis, and (7) micro-embolism.^18-20^

To our knowledge, no studies compare AKI and RRT in COVID-19 with other respiratory viruses that bind to or downregulate ACE2 and those viruses that are not associated with ACE2. This comparison enhances understanding of the role of ACE2 in the pathophysiology of AKI and could inform therapeutic strategies such as targeting of ACE2 with angiotensin converting enzyme inhibitors (ACEi) or angiotensin receptor blockers (ARBs) to mitigate AKI in COVID-19.

Accordingly, our objectives were to review and perform a meta-analysis of the frequencies of AKI and RRT in critically ill COVID-19 patients and compare those frequencies with critically ill patients who were infected by respiratory viral infections due to viruses that bind or downregulate ACE2 (ACE2-associated viruses) and viruses that do not involve ACE2 (non-ACE2-associated viruses).

## Methods

### Eligibility Criteria

We searched PubMed/MEDLINE and EMBASE from database inception up until March 25, 2021, for cohort studies, case control studies, and other systematic reviews and meta-analyses that reported the frequency of AKI, acute renal failure (ARF), or RRT in adults (18 years old or greater) infected with COVID-19. Full-text peer-reviewed articles and peer-reviewed articles published ahead of print were included. Letters and commentaries were also included if they provided patient characteristics. Only articles in English were included in the final analysis. Studies with less than 10 patients were excluded as were studies on cohorts of deceased patients and studies that reported only patients on dialysis or kidney transplant recipients. Non-peer-reviewed articles were excluded as were article abstracts, incomplete articles, research posters, conference abstracts, books, theses, and retracted articles.

### Search Strategy and Study Selection

We used a broad search strategy to find relevant articles using the following search terms: COVID, COVID-19, 2019-nCov, and SARS-CoV-2. These terms were combined using “AND” with the terms clinical features, clinical characteristics, kidney injury, renal, kidney, creatinine, renal replacement, and dialysis. For ACE2 and non-ACE2 associated viruses, we used similar search terms for kidney injury as above and replaced COVID-19 with these search terms: influenza, H1N1, H5N1, H3N2, H7N9, SARS, SARS-CoV-1, and MERS (see Supplemental Appendix for full search strategy details).

One reviewer (A.C.) screened titles and abstracts to identify reports of hospitalized COVID-19 patients with AKI or RRT. After reading the full texts of publications, studies were included if they reported the frequency of AKI, acute renal failure (ARF), or RRT in adults (18 years old or greater) infected with COVID-19. We similarly reviewed the literature on patients (18 years old or greater) infected with respiratory viruses that are associated with ACE2 through ACE2 binding or downregulation (SARS-CoV-1, influenza H1N1, influenza H7N9) and respiratory viruses that are not associated with ACE2 (e.g., Middle East Respiratory Syndrome Coronavirus [MERS-CoV] and other influenza strains besides H1N1, H7N9).^9,14-17,21^ Other studies were identified from the references in review articles and meta-analyses and were included if they met the inclusion criteria.

### Data Extraction

One reviewer (A.C.) extracted the studies from the literature in duplicate using a systematic template. We extracted the following data from each included study: author, journal of publication, study type, study location, age, sex, race, diabetes mellitus, hypertension, chronic kidney disease, shock, vasopressor use, intensive care unit (ICU) admission, ICU mortality, overall mortality, AKI, and RRT.AKI in the original publications was defined using a variety of definitions including the Kidney Disease Improving Global Outcomes (KDIGO) criteria, the Acute Kidney Injury Network (AKIN) criteria, the risk of renal failure, injury to the kidney, failure of kidney function, loss of kidney function, and end-stage renal failure (RIFLE) criteria or the presence of elevated serum creatinine.^22-24^ Critical illness was defined as admission to the intensive care unit (ICU). Results were not reported according to the Preferred Reporting Items for Systematic Reviews and Meta-Analyses (PRISMA) checklist, and a risk of bias assessment was performed on individual studies (Supplemental Table 4).

### Statistical Analysis

Results of all included studies were summarized quantitatively. Pooled estimates for the AKI and RRT rate within each virus group and comparison of rates between groups were obtained using random effects (RE) meta-analysis based on a random intercept logistic regression model with logit link and covariate being virus group.^25,26^ Pairwise comparisons between virus groups were done by testing the appropriate regression coefficient using Wald-type test from the estimated model. Mixed effects meta-regression was further conducted to adjust for the prevalence of shock and vasopressors use in each study when comparing the virus groups. Among the included studies, only overall prevalence of shock and vasopressors use was reported, but not the ones within critically ill. Since shock is a critical illness and vasopressors are only administered in critically ill patients, we will assume all patients who had shock or used vasopressors are critically ill patients for the purpose of computing these prevalence rates within critically ill. Given that few studies have both shock and vasopressors use data available, we examined these variables as two separate adjusted analyses. Log odds of AKI or RRT was considered as the outcome variable in the regression. Results from the meta-regression were thus expressed as odds ratios. Random effects meta-analysis and mixed effects meta-regression were performed using the meta package in R 3.6.2 (R Foundation for Statistical Computing, Vienna, Austria). Study heterogeneity was assessed visually by funnel plot with a minimum of 10 studies and asymmetry was assessed by the Begg adjusted rank correlation test. Statistical heterogeneity was assessed by I^2^.

## Results

### Study Selection and Characteristics

**Figure 1.**
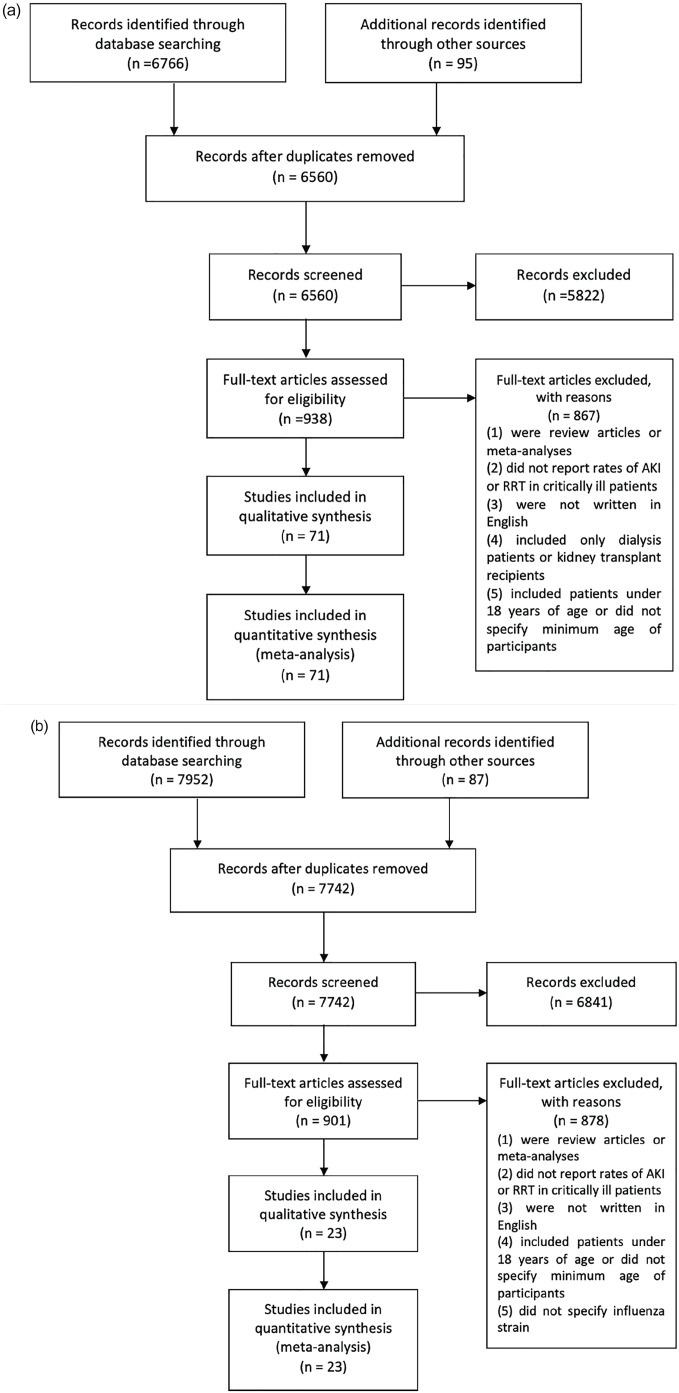
Flow diagrams of the search strategies showing included and excluded studies in the meta-analysis of acute kidney injury (AKI) and renal replacement therapy (RRT) in a) COVID-19 and b) ACE2- and non-ACE2-associated viral infections. *Note.* AKI = acute kidney injury; RRT = renal replacement therapy.

After screening 6560 COVID-19 titles and abstracts, 5622 studies were excluded. We assessed 938 full-text articles for their eligibility. Following assessment for eligibility, 867 COVID-19 articles were excluded because they (1) were review articles or meta-analyses, (2) did not report rates of AKI or RRT in critically ill patients, (3) were not written in English, (4) included only patients on dialysis or kidney transplant recipients, or (5) included patients under 18 years of age or did not specify the minimum age of study participants. After reviewing the full texts of 938 articles, 71 COVID-19 studies were included in the meta-analysis (Figure 1A).^1-3,13,27-94^ Similarly, 7742 articles on ACE2-associated (SARS-CoV-1, influenza H1N1, H7N9) and non-ACE2-associated viruses (MERS-CoV, other influenza strains) were screened based on their titles and abstracts and 6841 articles were excluded. The full-texts of 901 articles were reviewed and 20 studies on ACE2-associated viruses and 3 studies on non-ACE2-associated viruses were included in the meta-analysis (Figure 1B).^95-117^ The majority of the included COVID-19 studies were from the United States (n = 28) and China (n = 17) but other included studies were from England (n = 5), France (n = 4), Brazil (n = 3), South Korea (n = 2), Mexico (n = 2), and Canada, Sweden, Netherlands, Italy, Switzerland, Bahrain, India, United Arab Emirates, and Saudi Arabia (n = 1 each). The last study was conducted jointly in France and Spain.^38^ Included studies on ACE2-associated and non-ACE2-associated viruses were not predominantly associated with any country and were variable. The characteristics of the included studies are provided in Supplemental Table 1.

### Characteristics of Included Study Populations

Females made up 38% (n = 20 615) of the studied hospitalized COVID-19 population and represented 66% (n = 1082) and 32% (n = 120) of the populations infected with ACE2-associated viruses and non-ACE2-associated viruses, respectively (Supplemental Tables 1a-c). Of the 30 COVID-19 studies that reported race, 24% (n = 12 157) of the included population was Black or African American (Supplemental Table 1a). Similarly, 32% (n = 15 167), 27% (n = 8535), and 5% (n = 1163) of the included population were white, Hispanic or Latino, and Asian, respectively (data not shown). The frequency of hospitalized COVID-19 patients that had diabetes mellitus, hypertension, and chronic kidney disease (CKD) were 35% (n = 18 959), 58% (n = 27 072), and 13% (n = 6054), respectively (Supplemental Table 1a). In hospitalized patients infected with ACE2-associated viruses, 20% (n = 261), 31% (n = 159), and 6% (n = 69) had underlying diabetes mellitus, hypertension, and CKD, respectively (Supplemental Table 1b). Similarly, rates of these underlying comorbidities in hospitalized patients infected with non-ACE2-associated viruses were 50% (n = 170), 50% (n = 6), and 29% (n = 107), respectively (Supplemental Table 1c).

### Acute Kidney Injury in COVID-19 and in ACE2-Associated and Non-ACE2-Associated Viruses

**Figure 2.**
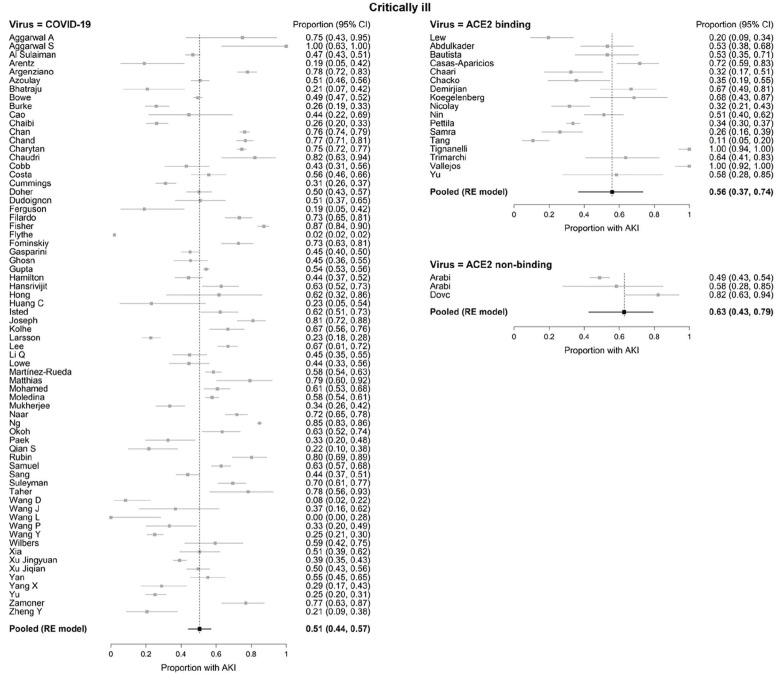
Forest plots of acute kidney injury (AKI) in critically ill patients with COVID-19 and ACE2- and non-ACE2-associated respiratory viral infections. *Note.* AKI = acute kidney injury; CI = confidence interval; RE = random effects; ACE2 = angiotensin converting enzyme 2.

Among hospitalized COVID-19 patients (n = 54 173), 44% were critically ill (n = 23 655) (Table 1). In patients hospitalized with ACE2-associated viruses (n = 1575), 94% (n = 1485) were critically ill whereas in hospitalized patients with non-ACE2-associated viruses (n = 370), 100% (n = 370) were critically ill. AKI frequencies did not differ between virus groups in critically ill patients: COVID-19 51% (95% confidence interval [CI]: 44%-57%), ACE2-associated respiratory viruses 56% (95% CI: 37%-74%, *P* = .610), and non-ACE2-associated viruses 63% (95% CI: 43%-79%, *P* = .255) or between ACE2-associated respiratory viruses and non-ACE2-associated viruses (*P* = .624) (Figure 2).

**Table 1.** AKI in Cohorts of Hospitalized Adult (≥18 Years Old) COVID-19 Patients.

Publication	Total (n)	ICU admission (n, % of total)	Shock (n, % of total)	Vasopressor use (n, % of total)	RRT (n, % of ICU)	RRT (n, % of total)	AKI (n, % of ICU)	AKI (n, % of total)	DIED + AKI (n, % of AKI)	DIED (n, % of ICU)	DIED (n, % of total)	KDIGO (Y/N)
Aggarwal et al^27^	32	12 (38)	6 (19)	6 (19)			9 (75)	13 (41)		9 (75)	9 (28)	X
Aggarwal et al^28^	16	8 (50)	8 (50)	8 (50)			8 (100)	11 (69)		3 (38)	3 (19)	
Al Sulaiman^29^	560	560 (100)		306 (55)	106 (19)	106 (195)	262 (47)	262 (47)		237 (42)	237 (42)	
Arentz et al^30^	21	21 (100)		14 (66)			4 (19)	4 (19)		11 (52)	11 (52)	X
Argenziano et al^31^	850	236 (28)		16 (2)	83 (35)	117 (14)	184 (78)	288 (34)		103 (44)	189 (22)	
Auld et al^32^	217	217 (100)	143 (66)	143 (66)	63 (29)	63 (29)				62 (29)	62 (29)	
Azoulay et al^33^	379	379 (100)		165 (44)	73 (19)	73 (19)	193 (51)	193 (51)		97 (26)	100 (26)	X
Bhatraju et al^34^	24	24 (100)	17 (71)	17 (71)	14 (58)	14 (58)	5 (21)	5 (21)		12 (50)	12 (50)	X
Bowe et al^35^	5216	1693 (32)				201 (4)	838 (49)	1655 (32)	559 (34)		832 (16)	X
Burke et al^36^	166	166 (100)			43 (26)	43 (26)	43 (26)	43 (26)	24 (56)	60 (36)	60 (36)	
Cao et al^37^	102	18 (18)	10 (10)		4 (22)	6 (6)	8 (44)	20 (20)	15 (75)	6 (33)	17 (17)	
Chaibi et al^38^	211	211 (100)		155 (73)	41 (19)	41 (19)	55 (26)	55 (26)	38 (69)	69 (33)	69 (33)	X
Chan et al^39^	3993	976 (24)		993 (25)	238 (24)	347 (9)	745 (76)	1835 (46)	918 (50)	324 (33)	1085 (27)	X
Chand et al^40^	300	300 (100)	233 (78)	233 (78)	133 (44)	133 (44)	230 (77)	230 (77)	138 (60)	157 (52)	157 (52)	X
Charytan et al^41^	4732	1056 (22)			213 (20)	237 (5)	788 (75)	1386 (29)	482 (35)	543 (51)	995 (21)	
Chaudri et al^42^	300	80 (27)				3/246 (1)	23/28 (82)	82 (27)	16/28 (57)		39 (13)	X
Cobb et al^43^	65	65 (100)	5 (8)	5 (8)	7 (11)	7 (11)	28 (43)	28 (43)		26 (40)	26 (40)	X
Costa et al^44^	102	102 (100)		48 (47)	27 (26)	27 (26)	57 (56)	57 (56)	19 (33)	23 (23)	23 (23)	X
Cummings et al^45^	257	257 (100)		170 (66)	79 (31)	79 (31)	80 (31)	80 (31)		101 (39)	101 (39)	X
Doher et al^46^	201	201 (100)	5/29 (17)	82 (41)	34 (17)	34 (17)	101 (50)	101 (50)	24 (24)	26 (13)	29 (14)	X
Dudoignon et al^47^	51	51 (100)			10 (20)	10 (20)	26 (51)	26 (51)	11 (42)	14 (27)	14 (27)	X
Ferguson et al^48 a^	72	21 (29)		13 (47)	4 (19)	4 (6)	4 (19)	4 (6)		3 (14)	6 (8)	X
Filardo et al^49^	270	135 (50)			55 (41)	56 (21)	99 (74)	123 (46)	65 (53)	72 (53)	78 (29)	X
Fisher et al^50^	3345	438/3341 (13)				164 (5)	382 (87)	1903 (57)			775 (23)	X
Flythe et al^51^	4264	4264 (100)	462 (11)	1773 (42)	82/4121 (2)	82/4121 (2)	82/4121 (2)	82/4121 (2)		1598 (37)	1598 (37)	
Fominskiy et al^52^	99	99 (100)			17 (17)	17 (17)	72 (72)	72 (72)	26 (36)	29 (29)	29 (29)	X
Gasparini et al^53^	372	372 (100)			121 (33)	121 (33)	168 (45)	168 (45)	81 (48)	139 (37)	139 (37)	X
Ghosn et al^54^	110	110 (100)		66 (60)	27 (25)	27 (25)	50 (45)	50 (45)	25 (50)	27 (25)	27 (25)	X
Gupta et al^55^	3099	3099 (100)		1305 (42)	637 (21)	637 (21)	1685 (54)	1685 (54)	403 (24)	1284 (41)	1284 (41)	X
Hamilton et al^56^	1032	165 (16)				32 (3)	73 (44)	210 (20)	110 (52)	59 (36)	326 (32)	X
Hansrivijit et al^57^	283	89 (31)		53 (19)		16 (6)	56 (63)	168 (59)	40 (24)		55 (19)	X
Hong et al^58^	98	13 (13)	9 (9)		3 (23)	3 (3)	8 (62)	9 (9)		4 (31)	5 (5)	X
Huang et al^1^	41	13 (32)	3 (7)		3 (23)	3 (7)	3 (23)	3 (7)		5 (38)	6 (15)	X
Isted et al^59^	85	85 (100)		51 (60)	17 (20)	17 (20)	53 (62)	53 (62)		38 (45)	38 (45)	X
Joseph et al^60^	100	100 (100)		51 (51)	13 (13)	13 (13)	81 (81)	81 (81)	28 (35)	29 (29)	29 (29)	X
Kolhe et al^61^	1161	96 (8)				23 (2)	64 (67)	304 (26)	184 (61)		419 (36)	X
Larsson et al^62^	260	260 (100)			59 (23)	59 (23)	59 (23)	59 (23)		60 (23)	60 (23)	X
Lee et al^63^	1002	274 (27)		261 (26)		59 (6)	183 (67)	294 (29)	118 (40)		172 (17)	X
Li et al^64^	107	107 (100)	38 (36)	38 (36)	20 (19)	20 (19)	48 (45)	48 (45)	40 (83)	51 (48)	51 (48)	X
Lowe et al^65^	81	81 (100)		49 (60)	16 (20)	16 (20)	36 (44)	36 (44)	9 (25)	12 (15)	12 (15)	X
Martínez-Rueda et al^66^	1170	443 (38)		110 (9)		50 (4)	259 (58)	349 (30)	182 (52)		321 (27)	X
Matthias et al^67^	188	29 (15)				11 (6)	23 (79)	41 (22)	11 (27)		19 (10)	X
Mitra et al^68^	117	117 (100)		65 (56)	16 (14)	16 (14)				18 (15)	18 (15)	
Mohamed et al^69^	575	173 (30)	148 (26)	148 (26)	77 (45)	89 (15)	105 (61)	161 (28)	80 (50)		80/575 (14)	X
Moledina et al^70^	2600	654 (25)		369 (14)	55 (8)	68 (3)	377 (58)	796 (31)	236 (30)		383 (15)	X
Mukherjee et al^71^	137	137 (100)			46 (34)	46 (34)	46 (34)	46 (34)			82 (60)	X
Naar et al^72^	206	206 (100)			46 (22)	46 (22)	148 (72)	148 (72)	19/46 (41)	19/46 (41)	19/46 (41)	X
Ñamendys-Silva et al^73^	164	164 (100)		139 (85)	24 (15)	24 (15)				85 (52)	85 (52)	
Ng et al^74^	9657	2409 (25)		2075 (21)	589 (24)	638 (7)	2042 (85)	3854 (40)	1997 (52)		2418 (25)	X
Okoh et al^75^	251	82 (33)	59 (24)		52 (63)	52 (21)	52 (63)	52 (21)	46 (88)	70 (85)	97 (39)	X
Paek et al^76^	704	46 (7)				8 (1)	15 (33)	28 (4)	13 (28)		24 (3)	X
Qian et al^77^	37	37 (100)	6 (16)		3 (8)	3 (8)	8 (22)	8 (22)		1 (3)	1 (3)	X
Rubin et al^78^	71	71 (100)		52 (73)	10/57 (18)	10/57 (18)	57 (80)	57 (80)	4/19 (21)	4 (6)	4 (6)	X
Samuel et al^79^	900	307 (34)	204 (23)	204 (23)			193 (63)	315 (35)		239 (78)	367 (41)	X
Sang et al^80^	210	210 (100)			52 (25)	52 (25)	92 (44)	92 (44)	65 (71)	93 (44)	93 (44)	X
Suleyman et al^81^	355	141 (40)	64 (18)	64 (18)	24 (17)	25 (7)	98 (70)	159 (45)		57 (40)	72 (20)	X
Taher et al^82^	73	23 (32)			7 (30)	7 (10)	18 (78)	29 (40)	12 (41)		13 (18)	X
Wang et al^2^	138	36 (26)	12 (9)	13 (9)	2 (6)	2 (1)	3 (8)	5 (4)		6 (17)	6 (4)	X
Wang et al^83^	116	19 (16)	2 (2)			5 (4)	7 (37)	12 (10)				X
Wang et al^84^	116	11 (9)				5 (4)	0 (0)	0 (0)		7 (64)	7 (6)	X
Wang et al^85^	45	45 (100)			6 (13)	6 (13)	15 (33)	15 (33)		19 (42)	19 (42)	
Wang et al^86^	344	344 (100)	114 (33)		9 (3)	9 (3)	86 (25)	86 (25)	80 (93)	133 (39)	133 (39)	X
Wilbers et al^87^	37	37 (100)			13 (35)	13 (35)	22 (59)	22 (59)	9 (41)	12 (32)	12 (32)	X
Xia et al^88^	81	81 (100)	51 (63)	63 (78)	8 (10)	8 (10)	41 (51)	41 (51)	34 (83)	60 (74)	60 (74)	X
Xu et al^89^	671	671 (100)		147 (22)	86 (13)	86 (13)	263 (39)	263 (39)	189 (72)	362 (54)	362 (54)	X
Xu et al^90^	239	239 (100)			12 (5)	12 (5)	119 (50)	119 (50)	99 (83)	147 (62)	147 (62)	X
Yan et al^91^	882	105 (12)	178 (20)			17 (2)	58 (55)	115 (13)	68 (59)		128 (15)	X
Yang et al^3^	52	52 (100)		18 (35)	9 (17)	9 (17)	15 (29)	15 (29)	12 (80)	32 (62)	32 (62)	X
Yu et al^92^	226	226 (100)	36 (16)		24 (11)	24 (11)	57 (25)	57 (25)		9 (4)	9 (4)	X
Zamoner et al^93^	101	52 (51)		47 (47)	24 (46)	25 (25)	40 (77)	51 (50)	33 (65)	34 (65)	37 (37)	X
Zheng et al^94^	34	34 (100)			5 (15)	5 (15)	7 (21)	7 (21)	0 (0)	0 (0)	0 (0)	X
Totals SARS-CoV-2	54173	23655 (44)	1813 (20)	9535 (29)	3441 (18)	4281 (8)	11159 (49)	18669 (35)	6562 (44)	6700 (40)	14227 (26)	—
Totals SARS-CoV-2 (KDIGO)	43257	16850 (39)	1200 (24)	7085 (26)	2801 (22)	3584 (8)	9769 (58)	16618 (38)	6041 (45)	3996 (40)	11064 (26)	—

*Note.* AKI = acute kidney injury; ICU = intensive care unit; RRT = renal replacement therapy; KDIGO = Kidney Disease Improving Global Outcomes Classification; SARS-CoV-2 = Severe Acute Respiratory Syndrome Coronavirus 2.

a Died or discharged to hospice.

Definitions of AKI varied between studies (Supplemental Table 2). After only including studies that defined AKI according to KDIGO criteria (61 studies, n = 43 257), the RE meta-analysis pooled AKI rate among critically ill COVID-19 patients was 52% (95% CI: 46%-58%).

Substantial statistical heterogeneity between studies was observed within each virus type (I^2^ = 98.1%, 83.9%, and 79.9% in critically ill patients for COVID-19, ACE2-associated, and non-ACE2-associated, respectively). The funnel plots (Figure 4) revealed no significant asymmetry (Begg test *P* > .05), but there seemed to exist some heterogeneity between studies. Four studies reported extreme AKI rate of either 100% or less than 1% (2 COVID-19 studies and 2 studies which examined ACE2-associated viruses).^28,84,110,112^ Sensitivity analysis which excluded these studies did not alter our conclusion for the comparisons between the 3 viruses.

### Renal Replacement Therapy in COVID-19 and in ACE2-Associated and Non-ACE2-Associated Viruses

**Figure 3.**
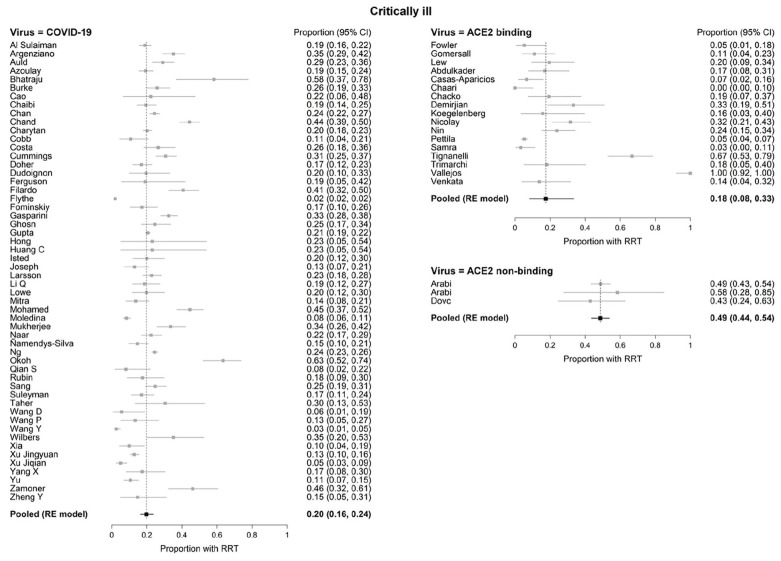
Forest plots of renal replacement therapy (RRT) in critically ill patients with COVID-19 and ACE2- and non-ACE2-associated respiratory viral infections. *Note.* CI = confidence interval; RE = random effects; RRT = renal replacement therapy; ACE2 = angiotensin converting enzyme 2.

**Figure 4.**
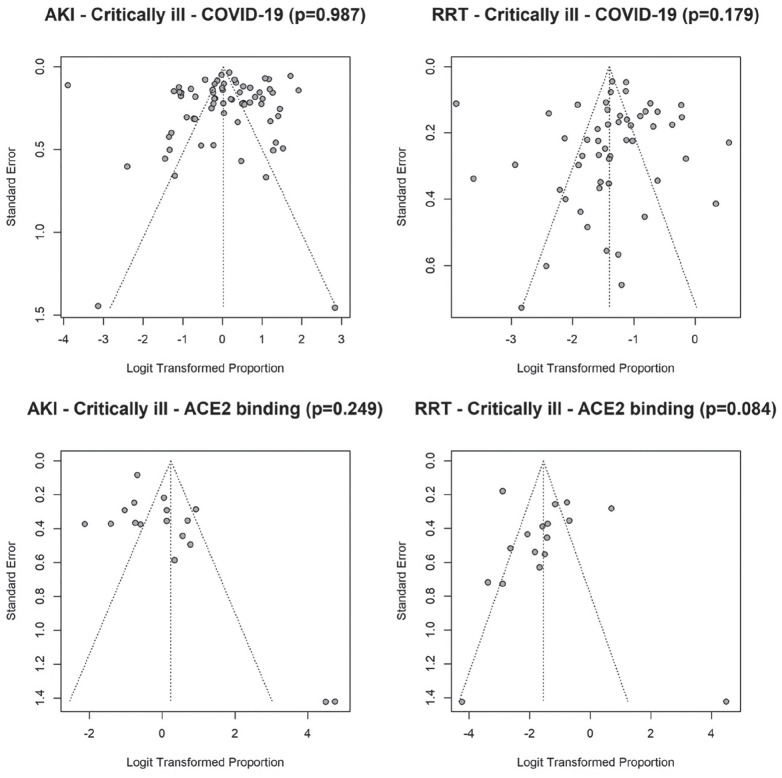
Funnel plots of acute kidney injury (AKI) and renal replacement therapy (RRT) in critically ill patients with COVID-19 and ACE2- and non-ACE2-associated respiratory viral infections. *Note.* AKI = acute kidney injury; RRT = renal replacement therapy; ACE2 = angiotensin converting enzyme 2.

In critically ill patients, there were no significant differences in the pooled RRT rates between COVID-19 (20%, 95% CI: 16%-24%) and ACE2-associated viruses (18%, 95% CI 8%-33%; *P* = .747). However, both COVID-19 and ACE2-associated viruses were significantly different (*P* < .001 for both) from non-ACE2-binding viruses (49%, 95% CI: 44%-54%; Figure 3). Substantial statistical heterogeneity between studies was observed within each virus type, except for non-ACE2-associated viruses (I^2^ = 95.4%, 89.6%, 0% in critically ill for COVID-19, ACE2-associated, and non-ACE2-associated, respectively). The funnel plots (Figure 4) revealed one outlying study on ACE2-associated viruses.^112^ Removal of this study did not alter our conclusion for the comparisons between the 3 groups of viruses.

After only including studies that defined AKI according to KDIGO criteria (61 studies, n = 43 257), RRT frequency among critically ill COVID-19 patients was 22% (n = 2801) (Table 1; RE meta-analysis pooled rate: 21% (95% CI: 17%-25%).

### Shock and Vasopressors in COVID-19 and Viruses That Are or Are not Associated With ACE2

In critically ill patients, shock occurred in 24% (n = 1813/6621) of COVID-19 patients, whereas vasopressors were used in 26% (n = 9535/17 170) of patients (Table 1). In cohorts of patients infected with ACE2-associated viruses, shock was seen in 25% (n = 76/299), whereas in patients infected with non-ACE2-associated viruses, 78% (n = 267/342) of patients experienced shock (Tables 2D and 3C). Similarly, in cohorts of patients infected with ACE2-associated viruses, vasopressors were initiated in 44% (n = 390/884) whereas 78% (n = 289/370) of patients infected with non-ACE2 associated viruses received vasopressor treatment (Tables 2D and 3C). After adjusting for differences in prevalence of shock or vasopressor use between studies, the AKI and RRT rates were not significantly different between the 3 viruses (Supplemental Table 3). Shock and vasopressor use were, however, both positively associated with AKI (odds ratio [OR]: 1.31, 95% CI: 1.14-1.49 and OR: 1.26, 95% CI: 1.07-1.48, respectively) and RRT use (OR: 1.36, 95% CI: 1.18-1.56 and OR: 1.26, 95% CI: 1.11-1.42, respectively) (Supplemental Table 3).

**Table 2.** AKI in Cohorts of Hospitalized Adult (≥18 Years Old) Patients Infected With Respiratory Viruses That Bind and/or Downregulate ACE2.

Publication	Total (n)	ICU admission (n, % of total)	Shock (n, % of total)	Vasopressor use (n, % of total)	RRT (% of ICU)	RRT (n, % of total)	AKI (n, % of ICU)	AKI (n, % of total)	DIED + AKI (n, % of AKI)	DIED (n, % ICU)	DIED (n, % of total)
(A) SARS-CoV-1											
Fowler et al^95a^	38	38 (100)		14 (37)	2 (5)	2 (5)				13 (34)	13 (34)
Gomersall et al^96^	54	54 (100)			6 (11)	6 (11)				14 (26)	14 (26)
Lew et al^97^	46	46 (100)	3 (7)		9 (20)	9 (20)	9 (20)	9 (20)		24 (52)	24 (52)
Totals SARS-CoV-1	138	138 (100)	3 (7)	14 (37)	17 (12)	17 (12)	9 (20)	9 (20)	n/a	51 (37)	51 (37)
(B) Influenza H1N1											
Abdulkader et al^98^	47	47 (100)		22 (47)	8 (17)	8 (17)	25 (53)	25 (53)	9 (36)	9 (19)	9 (19)
Bautista et al^99^	32	32 (100)					17 (53)	17 (53)	6 (35)		7 (22)
Casas-Aparicios et al^100^	60	60 (100)			4 (7)	4 (7)	43 (72)	43 (72)	17 (40)	19 (32)	19 (32)
Chaari^101^	34	34 (100)	4 (12)		0 (0)	0 (0)	11 (32)	11 (32)	7 (64)	11 (21)	11 (21)
Chacko et al^102^	31	31 (100)		18 (58)	6 (19)	6 (19)	11 (35)	11 (35)	4 (36)	6 (19)	6 (19)
Demirjian et al^103^	89	36 (40)			12 (33)	12 (13)	24 (67)	37 (42)	9 (24)	10 (28)	10/36 (28)
Koegelenberg et al^104^	19	19 (100)	8 (42)		3 (16)	3 (16)	13 (68)	13 (68)	12 (92)		13 (68)
Nicolay et al^105^	76	76 (100)		39 (51)	24 (32)	24 (32)	24 (32)	24 (32)		14 (18)	14 (18)
Nin et al^106^	84	84 (100)	37 (44)		20 (24)	20 (24)	43 (51)	43 (51)	30 (70)	46 (55)	46 (55)
Pettilä et al^107^	628	628 (100)		208/562 (37)	33 (5)	33 (5)	211 (34)	211 (34)	76 (36)	109 (17)	109 (17)
Samra et al^108^	61	61 (100)			2 (3)	2 (3)	16 (26)	16 (26)		50 (77)	50 (77)
Tang et al^109^	75	75 (100)	10 (13)				8 (11)	8 (11)		26 (35)	26 (35)
Tignanelli et al^110^	57	57 (100)		44 (77)	38 (67)	38 (67)	57 (100)	57 (100)	20 (35)	20 (35)	20 (35)
Trimarchi et al^111^	22	22 (100)			4 (18)	4 (18)	14 (64)	14 (64)	10 (71)	12 (55)	12 (55)
Vallejos et al^112^	44	44 (100)		35 (80)	44 (100)	44 (100)	44 (100)	44 (100)	36 (82)	36 (82)	36 (82)
Venkata et al^113^	66	29 (44)	10 (15)	10 (15)	4 (14)	4 (6)		14 (21)		5 (17)	5 (8)
Totals Influenza H1N1	1425	1335 (94)	69 (25)	376 (43)	202 (16)	202 (15)	561 (43)	588 (41)	236 (45)	373 (29)	393 (29)
(C) Influenza H7N9											
Yu et al^114^	12	12 (100)	4 (33)				7 (58)	7 (58)	5 (71)	6 (50)	6 (50)
Totals Influenza H7N9	12	12 (100)	4 (33)	n/a	n/a	n/a	7 (58)	7 (58)	5 (71)	6 (50)	6 (50)
(D) ACE2-associated virus totals											
SARS-CoV-1	138	138 (100)	3 (7)	14 (37)	17 (12)	17 (12)	9 (20)	9 (20)		51 (37)	51 (37)
Influenza H1N1	1425	1335 (94)	69 (25)	376 (43)	202 (16)	202 (15)	561 (43)	588 (41)	236 (45)	373 (29)	393 (29)
Influenza H7N9	12	12 (100)	4 (33)				7 (58)	7 (58)	5 (71)	6 (50)	6 (50)
Totals ACE2 viruses	1575	1485 (94)	76 (23)	390 (42)	219 (16)	219 (11)	577 (42)	604 (41)	241 (45)	430 (30)	450 (30)

*Note.* AKI = acute kidney injury; ACE2 = angiotensin converting enzyme 2; ICU = intensive care unit; RRT = renal replacement therapy; SARS-CoV-1 = Severe Acute Respiratory Syndrome Coronavirus 1.

a Vasopressor and inotrope use reported together.

**Table 3.** AKI in Cohorts of Hospitalized Adult (≥18 Years) Patients Infected With Respiratory Viral Infections That Do Not Bind or Downregulate ACE2.

Publication	Total (n)	ICU admission (n, % of total)	Shock (n, % of total)	Vasopressor use (n, % of total)	RRT (n, % of ICU)	RRT (n, % of total)	AKI (n, % of ICU)	AKI (n, % of total)	DIED + AKI (n, % of AKI)	DIED (n, % of ICU)	DIED (n, % of total)
(A) MERS-CoV											
Arabi et al^115^	12	12 (100)	5 (42)	8 (67)	7 (58)	7 (58)	7 (58)	7 (58)		7 (58)	7 (58)
Arabi et al^116^	330	330 (100)	262 (79)	262 (79)	161 (49)	161 (49)	161 (49)	161 (49)	131 (81)	217 (66)	217 (66)
Totals MERS-CoV	372	342 (100)	267 (78)	270 (79)	168 (49)	168 (49)	168 (49)	168 (49)	131 (81)	224 (65)	224 (65)
(B) Influenza (other than H1N1, H7N9)											
Dovč et al^117^	28	28 (100)		19 (68)	12 (43)	12 (43)	23 (82)	23 (82)	8 (35)	8 (29)	8 (29)
Totals Influenza (not H1N1 or H7N9)	28	28 (100)	n/a	19 (68)	12 (43)	12 (43)	23 (82)	23 (82)	8 (35)	8 (29)	8 (29)
(C) Non-ACE2-associated virus totals											
MERS-CoV	372	342 (100)	267 (78)	270 (79)	168 (49)	168 (49)	168 (49)	168 (49)	131 (81)	224 (65)	224 (65)
Influenza (not H1N1 or H7N9)	28	28 (100)		19 (68)	12 (43)	12 (43)	23 (82)	23 (82)	8 (35)	8 (29)	8 (29)
Totals Non ACE2 viruses	370	370 (100)	267 (78)	289 (78)	180 (49)	180 (49)	191 (52)	191 (52)	139 (76)	232 (63)	232 (63)

*Note.* AKI = acute kidney injury; ACE2 = angiotensin converting enzyme 2; ICU = intensive care unit; RRT = renal replacement therapy; MERS-CoV = Middle East Respiratory Syndrome Coronavirus.

## Discussion

Among critically ill patients, there were no differences in frequencies of AKI between COVID-19 patients and patients infected with other respiratory viruses. However, RRT rate was significantly lower in patients with COVID-19 or ACE2-associated viruses when compared with patients infected with non-ACE2 associated viruses. Lower frequencies of shock and use of vasopressors may partially account for lower RRT frequencies in COVID-19 and ACE2 associated viruses, because adjustments for shock and vasopressor use revealed no significant differences in rates of RRT between virus classes. CKD prevalence was similar in COVID-19 (13%) and in patients who had infection with ACE2-associated viruses (6%) but lower than in patients with non-ACE2-associated viruses (29%).

The similar rates of AKI in critically ill patients across viral groups suggests, but does not prove, that downregulation or binding of ACE2 may not be associated with increased rates of AKI. The AKI rates in COVID-19 in critically ill patients may not differ from other viruses because there may be common biological mechanisms by which COVID 19, other ACE2-associated and non-ACE2-associated viruses lead to AKI. Studies of renal structure and function in COVID-19 suggest several mechanisms of AKI. Post-mortem renal tissue of COVID-19 patients with AKI shows viral particles in the kidney and SARS-CoV-2 nucleoprotein antigen accumulation in renal tubules and in one study (n = 3), in all renal compartments.^118-120^ However, there is debate on whether these particles are indeed viral particles or other intracellular structures.^121^ Indications of diffuse proximal tubule injury, severe acute tubular necrosis, and lymphocyte infiltration have also been found.^118-120^ Evidence of renal tropism has also been found in cohorts of SARS, MERS, and influenza H1N1 patients.^122-124^ In one large study of patients with COVID-19-associated AKI, the most common cause of AKI was acute tubular necrosis, likely from hemodynamic instability possibly due to volume depletion, sepsis, or nephrotoxic drugs.^69^ Similarly, in another study, acute tubular injury (ATI) was the main post-mortem renal finding in 33 COVID-19 patients who had AKI.^125^ Post-mortem analysis of renal tissues of SARS and influenza H1N1 patients also revealed evidence of ATN.^124,126^

It is known that AKI and use of RRT are associated with an increased mortality rate.^127^ Here, we found that the AKI-associated mortality rate was 42% for COVID-19 patients, including those who were not critically ill, which is much less than what was seen in MERS patients (81%). Despite potential common pathophysiological mechanisms, this finding lends support to distinct pathophysiological processes of AKI, even among coronaviruses. The high frequencies of AKI and use of RRT in Middle East Respiratory Syndrome (MERS) may be due to the high renal expression of dipeptidyl peptidase-4, the viral receptor for MERS-CoV.^21^

A few other meta-analyses to date have also directly compared AKI and/or RRT rates among the different coronaviruses.^128,129^ In a meta-analysis comparing hospitalized patients infected with different coronaviruses and included studies until June 2020, the rates of AKI were found to be 9.0%, 9.6%, and 42.0% in COVID-19 patients, SARS patients, and MERS patients, respectively.^128^ In addition, urgent start kidney replacement therapy rates were reported to be 3.4% and 35.0% for COVID-19 and MERS patients, respectively. However, the study did not report rates of AKI or RRT in critically ill, or ICU, patients as we have done here.

With respect to mortality, it was found that among patients infected with different coronaviruses including SARS-CoV-2, the COVID-19 AKI mortality rate (78.0%) was lower than in SARS (86.6%) patients but higher than in MERS (68.5%) patients.^129^ Furthermore, another meta-analysis found that 72.3%, 98.9%, and 100% hospitalized patients with AKI who died had COVID-19, SARS, and MERS.^128^ The latter but not the former analysis is consistent with our findings that COVID-19-associated AKI mortality rates are lower than those in SARS or MERS-associated AKI (COVID-19 46%, SARS 90%, MERS 81%). Discrepancies in mortality rates across studies may be due in part to emerging evidence that suggests that AKI in COVID-19 has two distinct clinical phenotypes based on whether AKI presents early or late in patients during the course of illness.^130^ It has been suggested that different mechanisms underlie these phenotypes, each associated with an independent risk of mortality, with AKI-*early* possibly inciting multiple organ dysfunction whereas AKI-*late* may be a consequence of multiple organ dysfunction. Furthermore, AKI rates and AKI mortality appear to be much higher in the United States than in China, resulting in heterogeneity in AKI mortality rates among different studies.^131^

Variable definitions of AKI over these eras of COVID-19 versus ACE2-associated and non-ACE2-associated viruses may have masked differing rates of AKI across viruses, which may have emerged had one standard definition of AKI been used across all included studies. Earlier and better recognition and resuscitation may also have decreased the risks of AKI in the COVID-19 era compared with the earlier eras of infection by ACE2-associated and non-ACE2 associated viruses. Given the difference in mortality rates between groups (COVID-19 25%, patients infected with ACE2-associated viruses 30%, patients infected with non-ACE2-associated viruses 63%), it is possible that more patients in the non-ACE2 associated groups died before developing AKI, thereby underestimating true AKI rates among these patients.

Shock, such as septic shock due to bacterial sepsis, and use of vasopressors are common precedents and causes of AKI and need for RRT because of shock- and vasopressor-associated induced changes in total renal blood flow and distribution of renal blood flow as well as increased levels of pro-inflammatory cytokines and other mediators of AKI.^132-136^ In cohorts of critically ill patients with COVID-19, 84% and 70% of non-survivors had septic shock while 60% and 50% had AKI, respectively, suggesting that sepsis and septic shock contribute to AKI among critically ill COVID-19 patients.^12,86^ Septic AKI may also be due to pro-inflammatory cytokines which worsen AKI as well as angiotensin II because it is pro-inflammatory.^132-135^ Here, adjusting for shock and vasopressors using meta-regression analysis removed the significant differences in rate of RRT between virus classes suggesting—but not proving—that the lower rates of shock and use of vasopressors explains in part the lower rate of RRT in COVID-19 and ACE2-associated viruses compared with the non-ACE2-associated viruses.

Our study complements and extends a meta-analysis by Silver and colleagues that found similar rates of AKI (46%, 95% CI: 35%-57%) in critically ill COVID-19 patients but included fewer studies (because they only searched up until October 14, 2020, and excluded studies not using KDIGO criteria to define AKI), did not adjust for presence of shock or use of vasopressors, and did not compare rates of AKI and RRT in COVID-19 with those rates in patients infected with viruses that do or do not downregulate ACE2.^137^ Similarly, they found that kidney replacement therapy rates among critically ill COVID-19 patients was 19% (95% CI: 15%-22%).^137^ After including studies that defined AKI using KDIGO criteria, we found that AKI and RRT rates among hospitalized, critically ill COVID-19 patients were 52% and 22%, respectively.

The low prevalence (6%) of pre-existing CKD among cohorts of COVID-19 patients suggests that having CKD may not increase patient susceptibility to SARS-CoV-2 infection (Table 1). Use of ACEi or ARBs are cornerstones of CKD treatment and may have conferred a protective effect on these patients. However, the role of ACEi and ARBs in protecting from COVID-19 is controversial and unproven thus far. It is unclear whether developing AKI associated with COVID-19 alters the risk of developing CKD later in life.^138^

Our result that ACE2 association has a minimal impact on rates of AKI in hospitalized patients provides a rationale for trials of renin-angiotensin system inhibitors for the therapeutic management of COVID-19. Two recent randomized controlled trials of continuing or discontinuing ARBs in hospitalized patients already on ARBs found no difference in mortality between groups. However, we note that the mortality rates in the Brazil trial were very low (2.8% vs 2.7% continuing vs discontinuing ARBs, respectively) and therefore the trial was underpowered for such low rates.^139,140^

Our study has limitations which include the variable definitions for AKI used in different eras and in different cohorts. AKIN and RIFLE criteria were used to diagnose AKI in earlier SARS, influenza, and MERS studies whereas KDIGO was used in the majority of COVID-19 studies.^22-24^ While there is much overlap in the criteria, they are not identical, so some of the difference in AKI rates may be attributable to the different criteria used. We acknowledge that there is a possibility of overlap in reported cases as has been reported in other COVID-19 studies.^141^ Another limitation is that we could not match severity of infection or do propensity matching across studies. Other limitations include the high I^2^ value associated with COVID-19 studies (98.1%) and that only one reviewer screened the literature and extracted the data. Last, we did not include non-English publications. The inclusion of many studies from China aided our accurate reflection of AKI frequency in COVID-19.

## Conclusions

In conclusion, AKI frequencies were not significantly different between critically ill patients, that is, patients admitted to the ICU, with COVID-19 and other respiratory viral infections. However, the rate of RRT is lower in patients with COVID-19 or ACE2-associated viruses when compared with patients infected with non-ACE2-binding viruses, which might partly be due to the lower frequencies of shock and use of vasopressors in these two virus groups. However, despite being statistically significant, only 3 studies on non-ACE2-associated viruses were included in the meta-analysis and thus, more studies on non-ACE2-associated viruses are necessary to support this finding. Shock and vasopressor use may contribute to AKI and RRT in COVID-19. Lastly, underlying CKD rates were similar in COVID-19 and in patients who had infection with ACE2-associated and non-ACE2-associated viruses. Data from comprehensive prospective registries may elucidate true rates of AKI and RRT in COVID-19.

## Supplemental Material

sj-pdf-1-cjk-10.1177_20543581211052185 – Supplemental material for Acute Kidney Injury and Renal Replacement Therapy in COVID-19 Versus Other Respiratory Viruses: A Systematic Review and Meta-Analysis Click here for additional data file. Supplemental material, sj-pdf-1-cjk-10.1177_20543581211052185 for Acute Kidney Injury and Renal Replacement Therapy in COVID-19 Versus Other Respiratory Viruses: A Systematic Review and Meta-Analysis by A. Cau, M. P. Cheng, Terry Lee, A. Levin, T. C. Lee, D. C. Vinh, F. Lamontagne, J. Singer, K. R. Walley, S. Murthy, D. Patrick, O. Rewa, B. Winston, J. Marshall, J. Boyd and Russell JA in Canadian Journal of Kidney Health and Disease

## References

[1] Huang C Wang Y Li X , et al. Clinical features of patients infected with 2019 novel coronavirus in Wuhan, China. Lancet. 2020;395:497-506. doi:10.1016/s0140-6736(20)30183-5. 31986264 PMC7159299

[2] Wang D Hu B Hu C , et al. Clinical characteristics of 138 hospitalized patients with 2019 novel coronavirus-infected pneumonia in Wuhan, China. JAMA. 2020;323:1061-1069. doi:10.1001/jama.2020.1585. 32031570 PMC7042881

[3] Yang X Yu Y Xu J , et al. Clinical course and outcomes of critically ill patients with SARS-CoV-2 pneumonia in Wuhan, China: a single-centered, retrospective, observational study. Lancet Respir Med. 2020;8:475-481. doi:10.1016/S2213-2600(20)30079-5. 32105632 PMC7102538

[4] Hoffmann M Kleine-Weber H Schroeder S , et al. SARS-CoV-2 cell entry depends on ACE2 and TMPRSS2 and is blocked by a clinically proven protease inhibitor. Cell. 2020;181:271-280.e278. doi:10.1016/j.cell.2020.02.052. PMC7102627 32142651

[5] Walls AC Park YJ Tortorici MA , et al. Structure, function, and antigenicity of the SARS-CoV-2 spike glycoprotein. Cell. 2020;181:281-292.e286. doi:10.1016/j.cell.2020.02.058. PMC7102599 32155444

[6] Zhou P Yang X-L Wang X-G , et al. A pneumonia outbreak associated with a new coronavirus of probable bat origin. Nature. 2020;579:270-273. doi:10.1038/s41586-020-2012-7. 32015507 PMC7095418

[7] Gomes ERM Lara AA Almeida PWM , et al. Angiotensin-(1-7) prevents cardiomyocyte pathological remodeling through a nitric oxide/guanosine 3’,5’-cyclic monophosphate-dependent pathway. Hypertension. 2010;55:153-160. doi:10.1161/HYPERTENSIONAHA.109.143255. 19996065

[8] Paz Ocaranza M Riquelme JA García L , et al. Counter-regulatory renin–angiotensin system in cardiovascular disease. Nat Rev Cardiol. 2020;17:116-129. doi:10.1038/s41569-019-0244-8. 31427727 PMC7097090

[9] Li W Moore MJ Vasilieva N , et al. Angiotensin-converting enzyme 2 is a functional receptor for the SARS coronavirus. Nature. 2003;426:450-454. doi:10.1038/nature02145. 14647384 PMC7095016

[10] Crackower MA Sarao R Oudit GY , et al. Angiotensin-converting enzyme 2 is an essential regulator of heart function. Nature. 2002;417:822-828. doi:10.1038/nature00786. 12075344

[11] Hamming I Timens W Bulthuis M Lely AT Navis G van Goor H. Tissue distribution of ACE2 protein, the functional receptor for SARS coronavirus. A first step in understanding SARS pathogenesis. J Pathol. 2004;203:631-637. doi:10.1002/path.1570. 15141377 PMC7167720

[12] Zhou F Yu T Du R , et al. Clinical course and risk factors for mortality of adult inpatients with COVID-19 in Wuhan, China: a retrospective cohort study. Lancet. 2020;395:1054-1062. doi:10.1016/S0140-6736(20)30566-3. 32171076 PMC7270627

[13] Shi S Qin M Shen B , et al. Association of cardiac injury with mortality in hospitalized patients with COVID-19 in Wuhan, China. JAMA Cardiol. 2020;5:802-810. doi:10.1001/jamacardio.2020.0950. 32211816 PMC7097841

[14] Kuba K Imai Y Rao S , et al. A crucial role of angiotensin converting enzyme 2 (ACE2) in SARS coronavirus–induced lung injury. Nat Med. 2005;11:875-879. doi:10.1038/nm1267. 16007097 PMC7095783

[15] Liu X Yang N Tang J , et al. Downregulation of angiotensin-converting enzyme 2 by the neuraminidase protein of influenza A (H1N1) virus. Virus Res. 2014;185:64-71. doi:10.1016/j.virusres.2014.03.010. 24662240 PMC7114376

[16] Yang P Gu H Zhao Z , et al. Angiotensin-converting enzyme 2 (ACE2) mediates influenza H7N9 virus-induced acute lung injury. Sci Rep. 2014;4:7027. doi:10.1038/srep07027. 25391767 PMC4229671

[17] Zou Z Yan Y Shu Y , et al. Angiotensin-converting enzyme 2 protects from lethal avian influenza A H5N1 infections. Nat Commun. 2014;5:3594. doi:10.1038/ncomms4594. 24800825 PMC7091848

[18] Cantador E Núñez A Sobrino P , et al. Incidence and consequences of systemic arterial thrombotic events in COVID-19 patients. J Thromb Thrombolysis. 2020;50:543-547. doi:10.1007/s11239-020-02176-7. 32519165 PMC7282535

[19] McFadyen JD Stevens H Peter K. The emerging threat of (micro)thrombosis in COVID-19 and its therapeutic implications. Circul Res. 2020;127:571-587. doi:10.1161/CIRCRESAHA.120.317447. PMC7386875 32586214

[20] Ronco C Reis T Husain-Syed F. Management of acute kidney injury in patients with COVID-19. Lancet Resp Med. 2020;8:738-742. doi:10.1016/S2213-2600(20)30229-0. PMC7255232 32416769

[21] Yin Y Wunderink RG. MERS, SARS and other coronaviruses as causes of pneumonia. Respirology. 2018;23:130-137. doi:10.1111/resp.13196. 29052924 PMC7169239

[22] Bellomo R Ronco C Kellum JA Mehta RL Palevsky P , Acute Dialysis Quality Initiative Workgroup. Acute renal failure—definition, outcome measures, animal models, fluid therapy and information technology needs: the Second International Consensus Conference of the Acute Dialysis Quality Initiative (ADQI) Group. Crit Care. 2004;8(4):R204-R212. doi:10.1186/cc2872. PMC522841 15312219

[23] Khwaja A. KDIGO clinical practice guidelines for acute kidney injury. Nephron Clin Pract. 2012;120:c179-c184. 10.1159/000339789 22890468

[24] Mehta RL Kellum JA Shah SV , et al. Acute Kidney Injury Network: report of an initiative to improve outcomes in acute kidney injury. Crit Care. 2007;11(2):R31. doi:10.1186/cc5713. PMC2206446 17331245

[25] Schwarzer G Chemaitelly H Abu-Raddad LJ Rücker G. Seriously misleading results using inverse of Freeman-Tukey double arcsine transformation in meta-analysis of single proportions. Res Synth Methods. 2019;10(3):476-483. doi:10.1002/jrsm.1348. 30945438 PMC6767151

[26] Stijnen T Hamza TH Ozdemir P. Random effects meta-analysis of event outcome in the framework of the generalized linear mixed model with applications in sparse data. Stat Med. 2010;29:3046-3067. doi:10.1002/sim.4040. 20827667

[27] Aggarwal A Shrivastava A Kumar A Ali A. Clinical and epidemiological features of SARS-CoV-2 patients in SARI ward of a tertiary care centre in New Delhi. J Assoc Physicians India. 2020;68(7):19-26. 32602676

[28] Aggarwal S Garcia-Telles N Aggarwal G , et al. Clinical features, laboratory characteristics, and outcomes of patients hospitalized with coronavirus disease 2019 (COVID-19): early report from the United States. Diagnosis. 2020;7:91-96. doi:10.1515/dx-2020-0046. 32352401

[29] Al Sulaiman KA Aljuhani O Eljaaly K , et al. Clinical features and outcomes of critically ill patients with coronavirus disease 2019 (COVID-19): a multicenter cohort study. Int J Infect Dis. 2021;105:180-187. doi:10.1016/j.ijid.2021.02.037. 33601030 PMC7882917

[30] Arentz M Yim E Klaff L , et al. Characteristics and outcomes of 21 critically ill patients with COVID-19 in Washington State. JAMA. 2020;323:1612-1614. 32191259 10.1001/jama.2020.4326 PMC7082763

[31] Argenziano MG Bruce SL Slater CL , et al. Characterization and clinical course of 1000 patients with coronavirus disease 2019 in New York: retrospective case series. BMJ. 2020;369:m1996. doi:10.1136/bmj.m1996. PMC7256651 32471884

[32] Auld SC Caridi-Scheible M Blum JM , et al. ICU and ventilator mortality among critically ill adults with coronavirus disease 2019. Crit Care Med. 2020;48(9):e799-e804. doi:10.1097/CCM.0000000000004457. PMC7255393 32452888

[33] Azoulay E Fartoukh M Darmon M , et al. Increased mortality in patients with severe SARS-CoV-2 infection admitted within seven days of disease onset. Intensive Care Med. 2020;46(9):1714-1722. doi:10.1007/s00134-020-06202-3. 32780165 PMC7417780

[34] Bhatraju PK Ghassemieh BJ Nichols M , et al. Covid-19 in critically ill patients in the Seattle region—case series. N Engl J Med. 2020;382(21):2012-2022. 32227758 10.1056/NEJMoa2004500 PMC7143164

[35] Bowe B Cai M Xie Y Gibson AK Maddukuri G Al-Aly Z. Acute kidney injury in a national cohort of hospitalized US veterans with COVID-19. Clin J Am Soc Nephrol. 2021;16(1):14-25. doi:10.2215/CJN.09610620. PMC7792643 33199414

[36] Burke E Haber E Pike CW Sonti R. Outcomes of renal replacement therapy in the critically ill with COVID-19. Med Intensiva. 2021;45:325-331. doi:10.1016/j.medin.2021.02.004. PMC7891048 34629584

[37] Cao J Tu W-J Cheng W , et al. Clinical features and short-term outcomes of 102 patients with coronavirus disease 2019 in Wuhan, China. Clin Infect Dis. 2020;71(15):748-755. 32239127 10.1093/cid/ciaa243 PMC7184479

[38] Chaibi K Dao M Pham T , et al. Severe acute kidney injury in patients with COVID-19 and acute respiratory distress syndrome. Am J Respir Crit Care Med. 2020;202(9):1299-1301. doi:10.1164/rccm.202005-1524LE. 32866028 PMC7605189

[39] Chan L Chaudhary K Saha A , et al. AKI in hospitalized patients with COVID-19. J Am Soc Nephrol. 2021;32(1):151-160. doi:10.1681/ASN.2020050615. 32883700 PMC7894657

[40] Chand S Kapoor S Orsi D , et al. COVID-19-associated critical illness—report of the first 300 patients admitted to intensive care units at a New York City Medical Center. J Intensive Care Med. 2020;35(10):963-970. doi:10.1177/0885066620946692. 32812834

[41] Charytan DM Parnia S Khatri M , et al. Decreasing incidence of acute kidney injury in patients with COVID-19 critical illness in New York City. Kidney Int Rep. 2021;6(4):916-927. doi:10.1016/j.ekir.2021.01.036. 33558853 PMC7857986

[42] Chaudhri I Moffitt R Taub E , et al. Association of proteinuria and hematuria with acute kidney injury and mortality in hospitalized patients with COVID-19. Kidney Blood Press Res. 2020;45(6):1018-1032. doi:10.1159/000511946. 33171466

[43] Cobb NL Sathe NA Duan KI , et al. Comparison of clinical features and outcomes in critically ill patients hospitalized with COVID-19 versus influenza. Ann Am Thorac Soc. 2021;18(4):632-640. doi:10.1513/AnnalsATS.202007-805OC. 33183067 PMC8009008

[44] Costa RLD Sória TC Salles EF , et al. Acute kidney injury in patients with Covid-19 in a Brazilian ICU: incidence, predictors and in-hospital mortality. J Bras Nefrol. 2021;43(3):349-358. 33570081 10.1590/2175-8239-JBN-2020-0144 PMC8428632

[45] Cummings MJ Baldwin MR Abrams D , et al. Epidemiology, clinical course, and outcomes of critically ill adults with COVID-19 in New York City: a prospective cohort study. Lancet (London, England). 2020;395(10239):1763-1770. doi:10.1016/S0140-6736(20)31189-2. PMC7237188 32442528

[46] Doher MP Torres de Carvalho FR Scherer PF , et al. Acute kidney injury and renal replacement therapy in critically ill COVID-19 patients: risk factors and outcomes: a single-center experience in Brazil. Blood Purif. 2021;50:520-530. doi:10.1159/000513425. 33341806 PMC7801990

[47] Dudoignon E Moreno N Deniau B , et al. Activation of the renin-angiotensin-aldosterone system is associated with Acute Kidney Injury in COVID-19. Anaesth Crit Care Pain Med. 2020;39(4):453-455. 32565254 10.1016/j.accpm.2020.06.006 PMC7301818

[48] Ferguson J Rosser JI Quintero O , et al. Characteristics and outcomes of coronavirus disease patients under nonsurge conditions, Northern California, USA, March-April 2020. Emerg Infect Dis. 2020;26(8):1679-1685. doi:10.3201/eid2608.201776. 32407284 PMC7392471

[49] Filardo TD Khan MR Krawczyk N , et al. Comorbidity and clinical factors associated with COVID-19 critical illness and mortality at a large public hospital in New York City in the early phase of the pandemic (March-April 2020). PLoS ONE. 2020;15(11):e0242760. doi:10.1371/journal.pone.0242760. PMC7682848 33227019

[50] Fisher M Neugarten J Bellin E , et al. AKI in hospitalized patients with and without COVID-19: a comparison study. J Am Soc Nephrol. 2020;31(9):2145-2157. doi:10.1681/ASN.2020040509. 32669322 PMC7461660

[51] Flythe JE Assimon MM Tugman MJ , et al. Characteristics and outcomes of individuals with pre-existing kidney disease and COVID-19 admitted to intensive care units in the United States. Am J Kidney Dis. 2021;77(2):190-203. doi:10.1053/j.ajkd.2020.09.003. 32961244 PMC7501875

[52] Fominskiy EV Scandroglio AM Monti G , et al. Prevalence, characteristics, risk factors, and outcomes of invasively ventilated COVID-19 patients with acute kidney injury and renal replacement therapy. Blood Purif. 2021;50(1):102-109. doi:10.1159/000508657. 32659757 PMC7445373

[53] Gasparini M Khan S Patel JM , et al. Renal impairment and its impact on clinical outcomes in patients who are critically ill with COVID-19: a multicentre observational study. Anaesthesia. 2021;76(3):320-326. doi:10.1111/anae.15293. 33948938

[54] Ghosn M Attallah N Badr M , et al. Severe acute kidney injury in critically ill patients with COVID-19 admitted to ICU: incidence, risk factors, and outcomes. J Clin Med. 2021;10(6):1217. doi:10.3390/jcm10061217. 33804100 PMC7998509

[55] Gupta S Coca SG Chan L , et al. AKI treated with renal replacement therapy in critically ill patients with COVID-19. J Am Soc Nephrol. 2021;32(1):161-176. doi:10.1681/ASN.2020060897. 33067383 PMC7894677

[56] Hamilton P Hanumapura P Castelino L , et al. Characteristics and outcomes of hospitalised patients with acute kidney injury and COVID-19. PLoS ONE. 2020;15(11):e0241544. doi:10.1371/journal.pone.0241544. PMC7608889 33141867

[57] Hansrivijit P Gadhiya KP Gangireddy M Goldman JD. Risk factors, clinical characteristics, and prognosis of acute kidney injury in hospitalized COVID-19 patients: a retrospective cohort study. Medicines (Basel, Switzerland). 2021;8(1):4. doi:10.3390/medicines8010004. PMC7825666 33430296

[58] Hong KS Lee KH Chung JH , et al. Clinical features and outcomes of 98 patients hospitalized with SARS-CoV-2 infection in Daegu, South Korea: a brief descriptive study. Yonsei Med J. 2020;61:431-437. 32390367 10.3349/ymj.2020.61.5.431 PMC7214108

[59] Isted A McDonnell A Jones E Grundy T Jeyabraba S Ali T et al. Clinical characteristics and outcomes of 85 intensive care patients with Covid-19 in South London: A single centre observational study. J Intensive Care Soc. 2020;175114372097154. doi:10.1177/1751143720971541 10.1177/1751143720971541 PMC10427843 37593533

[60] Joseph A Zafrani L Mabrouki A Azoulay E Darmon M. Acute kidney injury in patients with SARS-CoV-2 infection. Ann Intens Care. 2020;10(1):117-117. doi:10.1186/s13613-020-00734-z. PMC7471244 32880774

[61] Kolhe NV Fluck RJ Selby NM Taal MW. Acute kidney injury associated with COVID-19: a retrospective cohort study. PLoS Med. 2020;17(10):e1003406. doi:10.1371/journal.pmed.1003406. PMC7598516 33125416

[62] Larsson E Brattström O Agvald-Öhman C , et al. Characteristics and outcomes of patients with COVID-19 admitted to ICU in a tertiary hospital in Stockholm, Sweden. Acta Anaesthesiol Scand. 2021;65(1):76-81. doi:10.1111/aas.13694. 32892337 PMC7756749

[63] Lee JR Silberzweig J Akchurin O , et al. Characteristics of acute kidney injury in hospitalized COVID-19 patients in an urban academic medical center. Clin J Am Soc Nephrol. 2021;16(2):284-286. doi:10.2215/CJN.07440520. 32948642 PMC7863636

[64] Li Q Zhang T Li F , et al. Acute kidney injury can predict in-hospital mortality in elderly patients with COVID-19 in the ICU: a single-center study. Clin Interv Aging. 2020;15:2095-2107. doi:10.2147/CIA.S273720. 33204075 PMC7666828

[65] Lowe R Ferrari M Nasim-Mohi M , et al. Clinical characteristics and outcome of critically ill COVID-19 patients with acute kidney injury: a single centre cohort study. BMC Nephrol. 2021;22(1):92. doi:10.1186/s12882-021-02296-z. 33722189 PMC7957445

[66] Martínez-Rueda AJ Álvarez RD Méndez-Pérez RA , et al. Community- and hospital-acquired acute kidney injury in COVID-19: different phenotypes and dismal prognosis. Blood Purif. 2021;50(6):931-941. doi:10.1159/000513948. 33744901 PMC8089414

[67] Diebold M Schaub S Landmann E Steiger J Dickenmann M. Acute kidney injury in patients with COVID-19: a retrospective cohort study from Switzerland. Swiss Med Wkly. 2021;151:w20482. 10.4414/smw.2021.20482 33706383

[68] Mitra AR Fergusson NA Lloyd-Smith E , et al. Baseline characteristics and outcomes of patients with COVID-19 admitted to intensive care units in Vancouver, Canada: a case series. CMAJ. 2020;192(26):E694-E701. 10.1503/cmaj.200794 PMC7828881 32461326

[69] Mohamed MM Lukitsch I Torres-Ortiz AE , et al. Acute kidney injury associated with coronavirus disease 2019 in urban New Orleans. Kidney360. 2020;1:614-622. 10.34067/KID.0002652020 PMC8815549 35372932

[70] Moledina DG Simonov M Yamamoto Y , et al. The association of COVID-19 with acute kidney injury independent of severity of illness: a multicenter cohort study. Am J Kidney Dis. 2021;77(4):490-499. doi:10.1053/j.ajkd.2020.12.007. 33422598 PMC7791318

[71] Mukherjee V Toth AT Fenianos M , et al. Clinical outcomes in critically ill coronavirus disease 2019 patients: a unique New York City public hospital experience. Crit Care Explor. 2020;2(8):e0188. doi:10.1097/CCE.0000000000000188. PMC7437795 32885172

[72] Naar L Langeveld K El Moheb M , et al. Acute kidney injury in critically-ill patients with COVID-19: a single-center experience of 206 consecutive patients. Ann Surg. 2020;272(4):e280-e281. 10.1097/SLA.0000000000004319 32932328

[73] Ñamendys-Silva SA Alvarado-Ávila PE Domínguez-Cherit G , et al. Outcomes of patients with COVID-19 in the intensive care unit in Mexico: a multicenter observational study. Heart Lung. 2021;50(1):28-32. doi:10.1016/j.hrtlng.2020.10.013. 33138974 PMC7577687

[74] Ng JH Hirsch JS Hazzan A , et al. Outcomes among patients hospitalized with COVID-19 and acute kidney injury. Am J Kidney Dis. 2021;77(2):204-215.e1. doi:10.1053/j.ajkd.2020.09.002. 32961245 PMC7833189

[75] Okoh AK Sossou C Dangayach NS , et al. Coronavirus disease 19 in minority populations of Newark, New Jersey. Int J Equity Health. 2020;19:93. 32522191 10.1186/s12939-020-01208-1 PMC7286208

[76] Paek JH Kim Y Park WY , et al. Severe acute kidney injury in COVID-19 patients is associated with in-hospital mortality. PLoS ONE. 2020;15(12):e0243528. doi:10.1371/journal.pone.0243528. PMC7725289 33296419

[77] Qian S-Z Hong W-D Lingjie M Chenfeng L Zhendong F Pan J-Y. Clinical characteristics and outcomes of severe and critical patients with 2019 novel coronavirus disease (COVID-19) in Wenzhou: a retrospective study. Front Med (Lausanne). 2020;7:552002. doi:10.3389/fmed.2020.552002. 33015108 PMC7500473

[78] Rubin S Orieux A Prevel R , et al. Characterization of acute kidney injury in critically ill patients with severe coronavirus disease 2019. Clin Kidney J. 2020;13(3):354-361. doi:10.1093/ckj/sfaa099. 32695326 PMC7314187

[79] Samuel A Mechineni A Aronow WS Ismail M Manickam R. A review of the characteristics and outcomes of 900 COVID-19 patients hospitalized at a Tertiary Care Medical Center in New Jersey, USA. Arch Med Sci Atheroscler Dis. 2020;5:e306-e312. doi:10.5114/amsad.2020.103039. PMC7885814 33644490

[80] Sang L Chen S Zheng X , et al. The incidence, risk factors and prognosis of acute kidney injury in severe and critically ill patients with COVID-19 in mainland China: a retrospective study. BMC Pulm Med. 2020;20(1):290. 33167955 10.1186/s12890-020-01305-5 PMC7649893

[81] Suleyman G Fadel RA Malette KM , et al. Clinical characteristics and morbidity associated with coronavirus disease 2019 in a series of patients in metropolitan Detroit. JAMA Netw Open. 2020;3:e2012270. 10.1001/jamanetworkopen.2020.12270 PMC7298606 32543702

[82] Taher A Alalwan AA Naser N Alsegai O Alaradi A. Acute kidney injury in COVID-19 pneumonia: a single-center experience in Bahrain. Cureus. 2020;12(8):e9693. doi:10.7759/cureus.9693. PMC7425827 32802627

[83] Wang J Wang Z Zhu Y , et al. Identify the risk factors of COVID-19-related acute kidney injury: a single-center, retrospective cohort study. Front Med (Lausanne). 2020;7:436. doi:10.3389/fmed.2020.00436. 32850917 PMC7399623

[84] Wang L Li X Chen H , et al. Coronavirus disease 19 infection does not result in acute kidney injury: an analysis of 116 hospitalized patients from Wuhan, China. Am J Nephrol. 2020;51:343-348. 32229732 10.1159/000507471 PMC7179524

[85] Wang P Tan X Li Q , et al. Extra-pulmonary complications of 45 critically ill patients with COVID-19 in Yichang, Hubei province, China: a single-centered, retrospective, observation study. Medicine. 2021;100(9):e24604. doi:10.1097/MD.0000000000024604. PMC7939178 33655925

[86] Wang Y Lu X Chen H , et al. Clinical course and outcomes of 344 intensive care patients with COVID-19. Am J Respir Crit Care Med. 2020;201:1430-1434. 32267160 10.1164/rccm.202003-0736LE PMC7258632

[87] Wilbers TJ Koning MV. Renal replacement therapy in critically ill patients with COVID-19: a retrospective study investigating mortality, renal recovery and filter lifetime. J Crit Care. 2020;60:103-105. doi:10.1016/j.jcrc.2020.07.025. 32795841 PMC7391167

[88] Xia P Wen Y Duan Y , et al. Clinicopathological features and outcomes of acute kidney injury in critically ill COVID-19 with prolonged disease course: a retrospective cohort. J Am Soc Nephrol. 2020;31(9):2205-2221. doi:10.1681/ASN.2020040426. 32826326 PMC7461691

[89] Xu J Yang X Yang L , et al. Clinical course and predictors of 60-day mortality in 239 critically ill patients with COVID-19: a multicenter retrospective study from Wuhan, China. Crit Care. 2020;24(1):394. 32631393 10.1186/s13054-020-03098-9 PMC7336107

[90] Xu J Xie J Du B Tong Z Qiu H Bagshaw SM. Clinical characteristics and outcomes of patients with severe COVID-19 induced acute kidney injury. J Intensive Care Med. 2020;36(3):319-326. doi:10.1177/0885066620970858. 33267722

[91] Yan Q Zuo P Cheng L , et al. Acute kidney injury is associated with in-hospital mortality in older patients with COVID-19. J Gerontol A Biol Sci Med Sci. 2021;76(3):456-462. doi:10.1093/gerona/glaa181. 32766817 PMC7454401

[92] Yu Y Xu D Fu S , et al. Patients with COVID-19 in 19 ICUs in Wuhan, China: a cross-sectional study. Crit Care. 2020;24:219. doi:10.1186/s13054-020-02939-x. 32410714 PMC7223395

[93] Zamoner W Santos CADS Magalhães LE de Oliveira PGS Balbi AL Ponce D. Acute kidney injury in COVID-19: 90 days of the pandemic in a Brazilian public hospital. Front Med (Lausanne). 2021;8:622577. doi:10.3389/fmed.2021.622577. 33634152 PMC7900413

[94] Zheng Y Sun L-J Xu M , et al. Clinical characteristics of 34 COVID-19 patients admitted to intensive care unit in Hangzhou, China. J Zhejiang Univ Sci B. 2020;21(5):378-387. 32425003 10.1631/jzus.B2000174 PMC7238397

[95] Fowler RA Lapinsky SE Hallett D , et al. Critically ill patients with severe acute respiratory syndrome. JAMA. 2003;290:367-373. 12865378 10.1001/jama.290.3.367

[96] Gomersall CD Joynt GM Lam P , et al. Short-term outcome of critically ill patients with severe acute respiratory syndrome. Intens Care Med. 2004;30:381-387. 10.1007/s00134-003-2143-y PMC7080204 14740156

[97] Lew TWK Kwek T-K Tai D , et al. Acute respiratory distress syndrome in critically ill patients with severe acute respiratory syndrome. JAMA. 2003;290:374-380. 12865379 10.1001/jama.290.3.374

[98] Abdulkader RC Ho YL de Sousa Santos S Caires R Arantes MF Andrade L. Characteristics of acute kidney injury in patients infected with the 2009 influenza A (H1N1) virus. Clin J Am Soc Nephrol. 2010;5(11):1916-1921. 20671226 10.2215/CJN.00840110 PMC3001779

[99] Bautista E Arcos M Jimenez-Alvarez L , et al. Angiogenic and inflammatory markers in acute respiratory distress syndrome and renal injury associated to A/H1N1 virus infection. Exp Mol Pathol. 2013;94(3):486-492. 23542734 10.1016/j.yexmp.2013.03.007

[100] Casas-Aparicio GA León-Rodríguez I Hernández-Zenteno RJ , et al. Aggressive fluid accumulation is associated with acute kidney injury and mortality in a cohort of patients with severe pneumonia caused by influenza A H1N1 virus. PLoS ONE. 2018;13:e0192592. doi:10.1371/journal.pone.0192592. PMC5813941 29447205

[101] Chaari A Dammak H Chtara K Bahloul M Bouaziz M. Acute kidney injury in critically ill A(H1N1)-infected patients: a study of the prognoses. J Ren Care. 2011;37:128-133. 21810194 10.1111/j.1755-6686.2011.00224.x

[102] Chacko J Gagan B Ashok E Radha M Hemanth HV. Critically ill patients with 2009 H1N1 infection in an Indian ICU. Indian J Crit Care Med. 2010;14:77-82. 20859491 10.4103/0972-5229.68220 PMC2936736

[103] Demirjian SG Raina R Bhimraj A , et al. 2009 influenza A infection and acute kidney injury: incidence, risk factors, and complications. Am J Nephrol. 2011;34(1):1-8. doi:10.1159/000328386. 21625080

[104] Koegelenberg CFN Irusen EM Cooper R , et al. High mortality from respiratory failure secondary to swine-origin influenza A (H1N1) in South Africa. QJM. 2010;103:319-325. 20219780 10.1093/qjmed/hcq022

[105] Nicolay N Callaghan MA Domegan LM , et al. Epidemiology, clinical characteristics and resource implications of pandemic (H1N1) 2009 in intensive care units in Ireland. Crit Care Resusc. 2010;12:255-261. 21143086

[106] Nin N Lorente JA Soto L , et al. Acute kidney injury in critically ill patients with 2009 influenza A (H1N1) viral pneumonia: an observational study. Intensive Care Med. 2011;37:768-774. doi:10.1007/s00134-011-2167-7. 21394630 PMC7095219

[107] Pettilä V Webb SAR Bailey M , et al. Acute kidney injury in patients with influenza A (H1N1) 2009. Intensive Care Med. 2011;37:763-767. doi:10.1007/s00134-011-2166-8. 21394631

[108] Samra T Pawar M Yadav A. Comparative evaluation of acute respiratory distress syndrome in patients with and without H1N1 infection at a tertiary care referral center. Indian J Anaesth. 2011;55:47-51. doi:10.4103/0019-5049.76602. 21431053 PMC3057245

[109] Tang X Du R Wang R , et al. Comparison of hospitalized patients with ARDS caused by covid-19 and H1N1. Chest. 2020;158(1):195-205. 32224074 10.1016/j.chest.2020.03.032 PMC7151343

[110] Tignanelli CJ Wiktor AJ Vatsaas CJ , et al. Outcomes of acute kidney injury in patients with severe ARDS due to influenza A (H1N1) pdm09 virus. Am J Crit Care. 2018;27:67-73. 29292278 10.4037/ajcc2018901

[111] Trimarchi H Greloni G Campolo-Girard V , et al. H1N1 infection and the kidney in critically ill patients. J Nephrol. 2010;23:725-731. 20349409

[112] Vallejos A Arias M Cusumano A , et al. Dialysis for acute kidney injury associated with influenza A (H1N1) infection. Saudi J Kidney Dis Transpl. 2013;24:527-533. 23640625 10.4103/1319-2442.111045

[113] Venkata C Sampathkumar P Afessa B. Hospitalized patients with 2009 H1N1 influenza infection: the Mayo Clinic experience. Mayo Clin Proc 2010;85:798-805. 20664021 10.4065/mcp.2010.0166 PMC2931615

[114] Yu L Wang Z Chen Y , et al. Clinical, virological, and histopathological manifestations of fatal human infections by avian influenza A(H7N9) virus. Clin Infect Dis. 2013;57(10):1449-1457. doi:10.1093/cid/cit541. 23943822

[115] Arabi YM Arifi AA Balkhy HH , et al. Clinical course and outcomes of critically ill patients with middle east respiratory syndrome coronavirus infection. Ann Intern Med. 2014;160:389-397. doi:10.7326/m13-2486 %m 24474051. 24474051 10.7326/M13-2486

[116] Arabi YM Al-Omari A Mandourah Y , et al. Critically ill patients with the middle east respiratory syndrome: a multicenter retrospective cohort study. Crit Care Med. 2017;45:1683-1695. doi:10.1097/CCM.0000000000002621. 28787295

[117] Dovč A Premru V Pečavar B , et al. Acute kidney injury in critically-ill adult patients with seasonal influenza infection. Clin Nephrol. 2017;88:18-21. doi:10.5414/cnp88fx05. 28669380

[118] Diao B Feng Z Wang C , et al. Human kidney is a target for novel severe acute respiratory syndrome coronavirus 2 (SARS-CoV-2) infection. MedRxiv 2020. 10.1038/s41467-021-22781-1 PMC8096808 33947851

[119] Puelles VG Lütgehetmann M Lindenmeyer MT , et al. Multiorgan and renal tropism of SARS-CoV-2. N Engl J Med. 2020;383:590-592. doi:10.1056/NEJMc2011400. 32402155 PMC7240771

[120] Su H Yang M Wan C , et al. Renal histopathological analysis of 26 postmortem findings of patients with COVID-19 in China. Kidney Int. 2020;98:219-227. doi:10.1016/j.kint.2020.04.003. 32327202 PMC7194105

[121] Miller SE Brealey JK. Visualization of putative coronavirus in kidney. Kidney Int. 2020;98:231-232. doi:10.1016/j.kint.2020.05.004. 32437764 PMC7206426

[122] Ding Y He L Zhang Q , et al. Organ distribution of severe acute respiratory syndrome (SARS) associated coronavirus (SARS-CoV) in SARS patients: implications for pathogenesis and virus transmission pathways. J Pathol. 2004;203(2):622-630. doi:10.1002/path.1560. 15141376 PMC7167761

[123] Alsaad KO Hajeer AH Al Balwi M , et al. Histopathology of Middle East respiratory syndrome coronovirus (MERS-CoV) infection: clinicopathological and ultrastructural study. Histopathology. 2018;72(3):516-524. doi:10.1111/his.13379. 28858401 PMC7165512

[124] Carmona F Carlotti AP Ramalho LN Costa RS Ramalho FS. Evidence of renal infection in fatal cases of 2009 pandemic influenza A (H1N1). Am J Clin Pathol. 2011;136(3):416-423. doi:10.1309/AJCP1Y6LLHWSKYHW. 21846917

[125] Santoriello D Khairallah P Bomback AS , et al. Postmortem kidney pathology findings in patients with COVID-19. J Am Soc Nephrol. 2020;31:2158-2167. doi:10.1681/ASN.2020050744. 32727719 PMC7461662

[126] Chu KH Tsang WK Tang CS , et al. Acute renal impairment in coronavirus-associated severe acute respiratory syndrome. Kidney Int. 2005;67:698-705. doi:10.1111/j.1523-1755.2005.67130.x. 15673319 PMC7112337

[127] Kaddourah A Basu RK Bagshaw SM , et al. Epidemiology of acute kidney injury in critically ill children and young adults. N Engl J Med. 2016;376:11-20. doi:10.1056/NEJMoa1611391. 27959707 PMC5322803

[128] Zhou S Xu J Xue C , et al. Coronavirus-associated kidney outcomes in COVID-19, SARS, and MERS: a meta-analysis and systematic review. Ren Fail. 2020;43:1-15. doi:10.1080/0886022x.2020.1847724. 33256491 PMC7717867

[129] Chen Y-T Shao S-C Lai EC-C , et al. Mortality rate of acute kidney injury in SARS, MERS, and COVID-19 infection: a systematic review and meta-analysis. Critic Care. 2020;24:439. doi:10.1186/s13054-020-03134-8. PMC7364133 32677972

[130] Peng S Wang HY Sun X , et al. Early versus late acute kidney injury among patients with COVID-19-a multicenter study from Wuhan, China. Nephrol Dial Transplant. 2020;35:2095-2102. doi:10.1093/ndt/gfaa288. 33275762 PMC7798799

[131] Fu EL Janse RJ de Jong Y , et al. Acute kidney injury and kidney replacement therapy in COVID-19: a systematic review and meta-analysis. Clin Kidney J. 2020;13:550-563. doi:10.1093/ckj/sfaa160. 32897278 PMC7467593

[132] Gomez H Ince C De Backer D , et al. A unified theory of sepsis-induced acute kidney injury: inflammation, microcirculatory dysfunction, bioenergetics, and the tubular cell adaptation to injury. Shock. 2014;41:3-11. doi:10.1097/SHK.0000000000000052. PMC3918942 24346647

[133] Murugan R Karajala-Subramanyam V Lee M , et al. Acute kidney injury in non-severe pneumonia is associated with an increased immune response and lower survival. Kidney Int. 2010;77:527-535. doi:10.1038/ki.2009.502. 20032961 PMC2871010

[134] Vaduganathan M Vardeny O Michel T , et al. Renin–angiotensin–aldosterone system inhibitors in patients with Covid-19. N Engl J Med. 2020;382:1653-1659. doi:10.1056/NEJMsr2005760. 32227760 PMC7121452

[135] Zarbock A Gomez H Kellum JA. Sepsis-induced acute kidney injury revisited: pathophysiology, prevention and future therapies. Curr Opin Crit Care. 2014;20:588-595. doi:10.1097/MCC.0000000000000153. 25320909 PMC4495653

[136] Bellomo R Kellum JA Ronco C , et al. Acute kidney injury in sepsis. Intens Care Med. 2017;43:816-828. doi:10.1007/s00134-017-4755-7. 28364303

[137] Silver SA Beaubien-Souligny W Shah PS , et al. The prevalence of acute kidney injury in patients hospitalized with COVID-19 infection: a systematic review and meta-analysis. Kidney Med. 2021;3(1):83-98. doi:10.1016/j.xkme.2020.11.008. 33319190 PMC7723763

[138] Hsu RK Hsu C-Y. The role of acute kidney injury in chronic kidney disease. Semin Nephrol. 2016;36:283-292. doi:10.1016/j.semnephrol.2016.05.005. 27475659 PMC4979984

[139] Lopes RD Macedo AVS de Barros E Silva PGM , et al. Effect of discontinuing vs continuing angiotensin-converting enzyme inhibitors and angiotensin II receptor blockers on days alive and out of the hospital in patients admitted with COVID-19: a randomized clinical trial. JAMA. 2021;325(3):254-264. doi:10.1001/jama.2020.25864. 33464336 PMC7816106

[140] Cohen JB Hanff TC William P , et al. Continuation versus discontinuation of renin-angiotensin system inhibitors in patients admitted to hospital with COVID-19: a prospective, randomised, open-label trial. Lancet Respir Med. 2021;9(3):275-284. doi:10.1016/S2213-2600(20)30558-0. 33422263 PMC7832152

[141] Bauchner H Golub RM Zylke J. Editorial concern-possible reporting of the same patients with COVID-19 in different reports. JAMA. 2020;323:1256. doi:10.1001/jama.2020.3980. 32176775

