# Supplementary material for: Acute Kidney Injury and Renal Replacement Therapy in COVID-19 Versus Other Respiratory Viruses: A Systematic Review and Meta-Analysis
Source: Can J Kidney Health Dis. 2021 Oct 30;8:20543581211052185. doi: 10.1177/20543581211052185 (PMC8558598; doi:10.1177/20543581211052185)
Supplement: sj-pdf-1-cjk-10.1177_20543581211052185 – Supplemental material for Acute Kidney Injury and Renal Replacement Therapy in COVID-19 Versus Other Respiratory Viruses: A Systematic Review and Meta-Analysis [file sj-pdf-1-cjk-10.1177_20543581211052185.pdf]

Acute Kidney Injury and Renal Replacement Therapy in COVID-19 Versus Other Respiratory Viruses – A Systematic Review and Meta-Analysis - Supplementary Data and Appendix

Cau A BSc, Cheng MP MD, Lee Terry PH D, Levin A MD, Lee TC MD,

Vinh DC MD, Lamontagne F MD, Singer J PH D, Walley KR MD,

Murthy S MD, Patrick D MD, Rewa O MD, Winston B MD,

Marshall J MD, Boyd J MD, Russell JA MD

on behalf of ARBs Corona I.

Supplemental Table 1 – Included studies in the meta-analysis

a) COVID-19 studies

Publication	Year	Journal	Study Size (n)	Location	Virus	Female (n, %)	Age	Black/African American (n, %)	Diabetes Mellitus (n, %)	Hypertension (n, %)	Chronic Kidney Disease (n, %)
Aggarwal A[27]	2020	Journal of the Association of Physicians of India	32	New Delhi, India	SARS-CoV-2	13 (41%)	Median (IQR): 54.5 (46.25-60)	-	16 (50%)	11 (34%)	0 (0%)
Aggarwal S[28]	2020	Diagnosis	16	Des Moines, USA	SARS-CoV-2	4 (25%)	Median (range): 67 (38-95)	-	5 (31%)	9 (56%)	6 (38%)
Al Sulaiman[29]	2021	International Journal of Infectious Diseases	560	Saudi Arabia	SARS-CoV-2	143 (25%)	Mean (SD): 60 (14.58)	-	323 (58%)	300 (54%)	52 (9%)
Arentz[30]	2020	Journal of the American Medical Association	21	King and Snohomish Counties, Washington, USA	SARS-CoV-2	10 (48%)	Mean (range): 70 (43-92)	-	7 (33%)	-	2 (10%)
Argenziano[31]	2020	British Medical Journal	850	New York City, USA	SARS-CoV-2	482 (57%)	Median (IQR): Hospital floor: 64 (51-77), ICU: 62 (52-72)	160 (19%)	473 (56%)	759 (89%)	164 (19%)

Publication	Year	Journal	Study Size (n)	Location	Virus	Female (n, %)	Age	Black/African American (n, %)	Diabetes Mellitus (n, %)	Hypertension (n, %)	Chronic Kidney Disease (n, %)
Auld[32]	2020	Critical Care Medicine	217	Atlanta, USA	SARS-CoV-2	98 (45%)	Median (IQR): 64 (54-73)	153 (71%)	99 (46%)	134 (62%)	58 (27%)
Azoulay[33]	2020	Intensive Care Medicine	379	Paris, France	SARS-CoV-2	87 (23%)	Median (IQR): 66 (53-68)	-	114 (30%)	186 (49%)	64 (17%)
Bhatraju[34]	2020	New England Journal of Medicine	24	Seattle, USA	SARS-CoV-2	9 (38%)	Mean (SD): 64 (18)	-	14 (58%)	-	5 (21%)
Bowe[35]	2021	Clinical Journal of the American Society of Nephrology	5216	USA Department of Veteran Affairs	SARS-CoV-2	308 (6%)	Median (IQR): 70 (61-76)	2308 (44%)	2537 (49%)	3985 (76%)	-
Burke[36]	2021	Medicina Intensiva	166	Washington DC, USA	SARS-CoV-2	68 (41%)	Mean (SD): 63 (14)	114 (69%)	72 (43%)	116 (70%)	46 (15%)

Publication	Year	Journal	Study Size (n)	Location	Virus	Female (n, %)	Age	Black/African American (n, %)	Diabetes Mellitus (n, %)	Hypertension (n, %)	Chronic Kidney Disease (n, %)
Cao[37]	2020	Clinical Infectious Diseases	102	Wuhan, China	SARS-CoV-2	49 (48%)	Median (IQR): 54 (37-67)	-	11 (11%)	28 (27%)	4 (4%)
Chaibi[38]	2020	American Journal of Respiratory Critical Care Medicine	211	France and Spain	SARS-CoV-2	139 (66%)	Mean (SD): 60 (11)	-	78 (37%)	107 (51%)	18 (8%)
Chan[39]	2021	Journal of the American Society of Nephrology	3993	New York, USA	SARS-CoV-2	1704 (43%)	Median (IQR): 64 (56-78)	1054 (26%)	1019 (26%)	1527 (38%)	420 (11%)
Chand[40]	2020	Journal of Intensive Care Medicine	300	New York, USA	SARS-CoV-2	118 (39%)	Mean (SD): 57.8 (12.2)	80 (27%)	134 (45%)	200 (67%)	39 (13%)
Charytan[41]	2021	Kidney International Reports	4732	New York, USA	SARS-CoV-2	2030 (43%)	Median (IQR): 65 (51-76)	686 (14%)	1646 (35%)	2738 (58%)	761 (16%)
Chaudri[42]	2020	Kidney and Blood Pressure Research	300	New York, USA	SARS-CoV-2	133 (44%)	Mean (SD): 59.3 (17.7)	23 (8%)	72 (24%)	133 (44%)	32 (11%)

Publication	Year	Journal	Study Size (n)	Location	Virus	Female (n, %)	Age	Black/African American (n, %)	Diabetes Mellitus (n, %)	Hypertension (n, %)	Chronic Kidney Disease (n, %)
Cobb[43]	2021	Annals of the American Thoracic Society	65	Washington, USA	SARS-CoV-2	19 (29%)	Mean (SD): 60.4 (15.7)	3 (5%)	26 (40%)	-	14 (22%)
Costa[44]	2021	Brazilian Journal of Nephrology	102	Rio de Janeiro, Brazil	SARS-CoV-2	42 (41%)	Mean (SD): 66.5 (15.7)	-	32 (31%)	55 (54%)	-
Cummings[45]	2020	The Lancet	257	New York, USA	SARS-CoV-2	86 (33%)	Median (IQR): 62 (51-72)	49 (19%)	92 (36%)	162 (63%)	37 (14%)
Doher[46]	2020	Blood Purification	201	São Paulo, Brazil	SARS-CoV-2	78 (39%)	Median (IQR): 64.0 (52.0-80.0)	-	64 (32%)	98 (49%)	-
Dudoignon[47]	2020	Anesthesia Critical Care and Pain Medicine	51	Paris, France	SARS-CoV-2	12 (24%)	Median (IQR): 63 (57-69)	-	20 (39%)	31 (61%)	-
Ferguson[48]	2020	Emerging Infectious Diseases	72	California, USA	SARS-CoV-2	34 (47%)	Median (IQR): 60.4 (43.4-70.6)	4 (6%)	20 (28%)	26 (36%)	9 (13%)

Publication	Year	Journal	Study Size (n)	Location	Virus	Female (n, %)	Age	Black/African American (n, %)	Diabetes Mellitus (n, %)	Hypertension (n, %)	Chronic Kidney Disease (n, %)
Filardo[49]	2020	PLoS One	270	New York, USA	SARS-CoV-2	88 (33%)	Median (IQR): 58 (50-67)	29 (11%)	42 (16%)	-	12 (4%)
Fisher[50]	2020	Journal of the American Society of Nephrology	3345	New York, USA	SARS-CoV-2	1569 (47%)	Mean (SD): 64.4 (16.4)	1201 (36%)	906 (27%)	-	409 (12%)
Flythe[51]	2021	American Journal of Kidney Diseases	4264	USA (data from STOP-COVID study)	SARS-CoV-2	1550 (365)	Median (IQR): Maintenance HD: 65 (56-71); Non-dialysis dependent CKD: 69 (60-76); No CKD: 61 (51-70)	1266 (30%)	1763 (41%)	2612 (61%)	664 (16%)
Fominskiy[52]	2020	Blood Purification	99	Milan, Italy	SARS-CoV-2	19 (19%)	Median IQR: AKI: 63.0 (58.5-70.0); No AKI: 54.5 (46.0-66.2)	-	16 (16%)	42 (42%)	6 (6%)
Gasparini[53]	2020	Anaesthesia	372	London, England	SARS-CoV-2	103	Median (IQR): 59 (51-65)	48 (13%)	-	-	41 (11%)

Publication	Year	Journal	Study Size (n)	Location	Virus	Female (n, %)	Age	Black/African American (n, %)	Diabetes Mellitus (n, %)	Hypertension (n, %)	Chronic Kidney Disease (n, %)
Ghosn[54]	2021	Journal of Clinical Medicine	110	Abu Dhabi, United Arab Emirates	SARS-CoV-2	12 (11%)	Median (IQR): 50 (41-59)	-	42 (38%)	39 (35%)	6 (5%)
Gupta[55]	2021	Journal of the American Society of Nephrology	3099	United States (67 hospitals)	SARS-CoV-2	1096 (35%)	Median (IQR): 62 (51-71)	952 (31%)	1230 (40%)	1869 (60%)	897 (29%)
Hamilton[56]	2020	PLoS One	1032	Manchester, England	SARS-CoV-2	463 (45%)	Median (IQR): 71 (56-83)	94 (9%)	273 (26%)	-	144 (14%)
Hansrivijit[57]	2021	Medicines	283	Pennsylvania, USA	SARS-CoV-2	124 (44%)	Mean (SD): 64.1 (15.9)	88 (31%)	108 (38%)	189 (67%)	66 (23%)
Hong[58]	2020	Yonsei Medical Journal	98	Daegu, South Korea	SARS-CoV-2	60 (61%)	Mean (SD): 55.4 (17.1)	-	9 (9%)	30 (31%)	-
Huang C[1]	2020	The Lancet	41	Wuhan, China	SARS-CoV-2	11 (27%)	Median (IQR): 49* (41-58)	-	8 (20%)	6 (15%)	-
Isted[59]	2020	Journal of the Intensive Care Society	85	London, England	SARS-CoV-2	21 (25%)	Median (IQR): 57.3 (49.4-64.2)	37 (44%)	29 (34%)	44 (52%)	11 (13%)

Publication	Year	Journal	Study Size (n)	Location	Virus	Female (n, %)	Age	Black/African American (n, %)	Diabetes Mellitus (n, %)	Hypertension (n, %)	Chronic Kidney Disease (n, %)
Joseph[60]	2020	Annals of Intensive Care	100	Paris, France	SARS-CoV-2	30 (30%)	Median (IQR): 59 (53-67)	-	30 (30%)	56 (56%)	29 (29%)
Kolhe[61]	2020	PLoS One	1161	Derby, England	SARS-CoV-2	504 (43%)	Mean (SD): AKI: 74.9 (12.8); No AKI: 71.1 (17.0)	21 (2%)	255 (22%)	-	224 (19%)
Larsson[62]	2021	Acta Anaesthesiologica Scandinavica	260	Stockholm, Sweden	SARS-CoV-2	52 (20%)	Median (IQR): 59 (51-65)	-	68 (26%)	103 (40%)	4 (2%)
Lee[63]	2021	Clinical Journal of the American Society of Nephrology	1002	New York, USA	SARS-CoV-2	383 (38%)	Median (IQR): 66 (53-76)	119 (12%)	378 (38%)	597 (60%)	138 (14%)
Li Q[64]	2020	Clinical Interventions in Aging	107	Beijing, China	SARS-CoV-2	38 (36%)	Median (IQR): AKI: 73 (67-81); No AKI: 68 (63-75)	-	22 (21%)	73 (68%)	5 (5%)
Lowe[65]	2021	BMC Nephrology	81	Southampton, England	SARS-CoV-2	31 (38%)	Median (IQR): 57 (18)	7 (9%)	21 (26%)	30 (37%)	5 (6%)

Publication	Year	Journal	Study Size (n)	Location	Virus	Female (n, %)	Age	Black/African American (n, %)	Diabetes Mellitus (n, %)	Hypertension (n, %)	Chronic Kidney Disease (n, %)
Martínez-Rueda[66]	2021	Blood Purification	1170	Mexico City, Mexico	SARS-CoV-2	440 (38%)	Median (IQR): 53 (44-64)	-	329 (28%)	335 (29%)	36 (3%)
Matthias[67]	2021	Swiss Medical Weekly	188	Basel, Switzerland	SARS-CoV-2	73 (39%)	Median (IQR): 62 (48-73)	-	35 (19%)	86 (46%)	28 (15%)
Mitra[68]	2020	Canadian Medical Association Journal	117	Vancouver, Canada	SARS-CoV-2	38 (32%)	Median (IQR): 69 (60-75)	-	36 (31%)	54 (46%)	15 (13%)
Mohamed[69]	2020	Kidney360	575	New Orleans, USA	SARS-CoV-2	263 (46%)	Median (range): AKI: 65 (34-96); No AKI: 66 (23-97)	414 (72%)	281 (49%)	424 (74%)	172 (30%)
Moledina[70]	2021	American Journal of Kidney Diseases	2600	Connecticut, USA	SARS-CoV-2	1280 (49%)	Median (IQR): 65.6 (52.5-79.6)	646 (25%)	997 (38%)	1658 (64%)	426 (16%)
Mukherjee[71]	2020	Critical Care Explorations	137	New York, USA	SARS-CoV-2	38 (28%)	Median (IQR): 59 (51-70)	23 (17%)	51 (37%)	70 (51%)	20 (15%)

Publication	Year	Journal	Study Size (n)	Location	Virus	Female (n, %)	Age	Black/African American (n, %)	Diabetes Mellitus (n, %)	Hypertension (n, %)	Chronic Kidney Disease (n, %)
Naar[72]	2020	Annals of Surgery	206	Boston, USA	SARS-CoV-2	72 (35%)	Median: 60 (47-71)	23 (11%)	89 (43%)	103 (50%)	27 (13%)
Ñamendys-Silva[73]	2021	Heart and Lung	164	Mexico	SARS-CoV-2	50 (30%)	Mean (SD): 57.3 (13.7)	-	53 (32%)	63 (38%)	6 (4%)
Ng[74]	2021	American Journal of Kidney Diseases	9657	New York, USA	SARS-CoV-2	3910 (40%)	Median (IQR): AKI Stage 1-3: 71 (61-81); AKI dialysis: 64 (57-72); No AKI: 62 (51-75)	1910 (20%)	3469 (40%)	5730 (59%)	492 (5%)
Okoh[75]	2020	International Journal for Equity in Health	251	Newark, USA	SARS-CoV-2	122 (49%)	Median (IQR): 62 (49-74)	210 (84%)	115 (46%)	175 (70%)	46 (18%)
Paek[76]	2020	PLoS One	704	Daegu, South Korea	SARS-CoV-2	494 (70%)	Mean (SD): 57.7 (17.6)	-	123 (17%)	226 (32%)	-

Publication	Year	Journal	Study Size (n)	Location	Virus	Female (n, %)	Age	Black/African American (n, %)	Diabetes Mellitus (n, %)	Hypertension (n, %)	Chronic Kidney Disease (n, %)
Qian[77]	2020	Frontiers in Medicine	37	Wenzhou, China	SARS-CoV-2	11 (30%)	Mean (range): 57 (21-93)	-	8 (21%)	14 (37%)	1 (3%)
Rubin[78]	2020	Clinical Kidney Journal	71	Bordeaux, France	SARS-CoV-2	16 (23%)	Mean (SD): 61.2 (12.2)	-	21 (30%)	43 (61%)	4 (6%)
Samuel[79]	2021	Archives of Medical Science Atherosclerotic Diseases	900	New Jersey, USA	SARS-CoV-2	371 (41%)	Median: 64	175 (19%)	344 (38%)	518 (58%)	91 (10%)
Sang[80]	2020	BMC Pulmonary Medicine	210	Guangzhou, China	SARS-CoV-2	79 (38%)	Median (IQR): 64 (56-71)	-	44 (21%)	98 (47%)	10 (5%)
Suleyman[81]	2020	Journal of the American Medical Association	355	Detroit, USA	SARS-CoV-2	190 (54%)	Mean (SD): 61.5 (15.4)	260 (73%)	156 (44%)	258 (73%)	161 (45%)
Taher[82]	2020	Cureus	73	Manama, Bahrain	SARS-CoV-2	29 (40%)	Mean (SD): 54.3 (13.5)	-	33 (45%)	31 (43%)	6 (8%)
Wang D[2]	2020	Journal of the American Medical Association	138	Wuhan, China	SARS-CoV-2	63 (46%)	Median (IQR): 56 (42-68)	-	14 (10%)	43 (31%)	4 (3%)

Publication	Year	Journal	Study Size (n)	Location	Virus	Female (n, %)	Age	Black/African American (n, %)	Diabetes Mellitus (n, %)	Hypertension (n, %)	Chronic Kidney Disease (n, %)
Wang J[83]	2020	Frontiers in Medicine	116	Wuhan, China	SARS-CoV-2	54 (47%)	Median (IQR): 62 (55-69)	-	20 (17%)	47 (41%)	-
Wang L[84]	2020	American Journal of Nephrology	116	Wuhan, China	SARS-CoV-2	49 (42%)	Median (IQR): 54 (38-69)	-	18 (16%)	43 (37%)	5 (4%)
Wang P[85]	2021	Medicine	45	Hubei, China	SARS-CoV-2	15 (33%)	Median (IQR): 64 (47-71)	-	12 (27%)	-	3 (7%)
Wang Y[86]	2020	American Journal of Respiratory and Critical Care Medicine	344	Wuhan, China	SARS-CoV-2	165 (48%)	Median (IQR): 64 (52-72)	-	64 (19%)	141 (41%)	-
Wilbers[87]	2020	Journal Critical Care	37	Arnhem, The Netherlands	SARS-CoV-2	10 (27%)	Median (IQR): AKI-RRT: 64 (42-73); AKI no RRT: 71 (60-76); No AKI: 68 (42-81)	-	4 (11%)	10 (27%)	3 (8%)

Publication	Year	Journal	Study Size (n)	Location	Virus	Female (n, %)	Age	Black/African American (n, %)	Diabetes Mellitus (n, %)	Hypertension (n, %)	Chronic Kidney Disease (n, %)
Xia[88]	2020	Journal of the American Society of Nephrology	81	Wuhan, China	SARS-CoV-2	27 (33%)	Mean (SD): 66.6 (11.4)	-	19 (24%)	43 (53%)	3 (4%)
Xu Jiqian[89]	2020	Critical Care	239	Wuhan, China	SARS-CoV-2	96 (40%)	Mean (SD): 62.5 (13.3)	-	44 (18%)	105 (44%)	-
Xu Jingyuan[90]	2021	Journal of Intensive Care Medicine	671	China	SARS-CoV-2	237 (35%)	Median (IQR): 65 (56-73)	-	131 (20%)	287 (43%)	-
Yan[91]	2020	Journal of Gerontology: Biomedical Sciences	882	Wuhan, China	SARS-CoV-2	442 (50%)	Median (IQR): 71 (68-77)	-	277 (31%)	-	83 (9%)
Yang X[3]	2020	The Lancet	52	Wuhan, China	SARS-CoV-2	17 (33%)	Mean (SD): 59.7 (13.3)	-	9 (17%)	-	-
Yu[92]	2020	Critical Care	226	Wuhan, China	SARS-CoV-2	87 (39%)	Median (IQR): 64 (57-70)	-	47 (21%)	96 (42%)	8 (4%)
Zamoner[93]	2021	Frontiers in Medicine	101	São Paulo, Brazil	SARS-CoV-2	46 (46%)	Mean (SD): 55 (54.4)	-	34 (34%)	54 (53%)	10 (10%)

Publication	Year	Journal	Study Size (n)	Location	Virus	Female (n, %)	Age	Black/African American (n, %)	Diabetes Mellitus (n, %)	Hypertension (n, %)	Chronic Kidney Disease (n, %)
Zheng[94]	2020	Journal of Zhejiang University Science	34	Hangzhou, China	SARS-CoV-2	11 (32%)	Median (IQR): 66 (58-76)	-	8 (24%)	22 (65%)	2 (6%)

b) ACE2-associated viruses

Publication	Year	Journal	Study Size (n)	Location	Virus	Female (n, %)	Age	Diabetes Mellitus (n, %)	Hypertension (n, %)	Chronic Kidney Disease (n, %)
Fowler[95]	2003	Journal of the American Medical Association	38	Toronto, Canada	SARS-CoV-1	15 (39%)	Median (IQR): 57.4 (39.0-69.6)	14 (37%)	4 (11%)	3 (8%)
Gomersall[96]	2004	Journal of the American Medical Association	54	Hong Kong	SARS-CoV-1	23 (43%)	Mean (SD): 49.6 (16.9)	-	-	-
Lew[97]	2003	Journal of the American Medical Association	46	Singapore	SARS-CoV-1	22 (48%)	Median (range): 51 (20-78)	7 (15%)	14 (30%)	3 (7%)
Abdulkader[98]	2010	Clinical Journal of the American Society of Nephrology	47	São Paulo, Brazil	H1N1	25 (53%)	Mean (SD): 43 (15)	5 (11%)	6 (13%)	5 (11%)

Publication	Year	Journal	Study Size (n)	Location	Virus	Female (n, %)	Age	Diabetes Mellitus (n, %)	Hypertension (n, %)	Chronic Kidney Disease (n, %)
Bautista[99]	2013	Experimental and Molecular Pathology	32	Mexico City, Mexico	H1N1	10 (31%)	Mean (SD): ARDS/AKI group: 41.4 (11.8), ARDS only: 39.6 (11.5)	-	9 (28%)	-
Casas-Aparicios[100]	2018	PLOS One	60	Mexico City, Mexico	H1N1	21 (35%)	Median (IQR): 47.5 (44.2-50.9)	5 (8%)	13 (22%)	-
Chaari[101]	2011	Journal of Renal Care	34	Sfax, Tunisia	H1N1	15 (44%)	Mean (SD): 37.29 (20.81)	-	-	-
Chacko[102]	2010	Indian Journal of Critical Care Medicine	31	Karnataka State, India	H1N1	13 (42%)	Median (IQR): 35 (28.2-42.8)	3 (10%)	4 (13%)	-
Demirjian[103]	2011	American Journal of Nephrology	89	Cleveland, USA	H1N1	40 (45%)	Mean (SD): AKI: 49 (12), No AKI: 46 (16.3)	31 (86%)	-	16 (18%)

Publication	Year	Journal	Study Size (n)	Location	Virus	Female (n, %)	Age	Diabetes Mellitus (n, %)	Hypertension (n, %)	Chronic Kidney Disease (n, %)
Koegelenberg[104]	2010	QJM: An International Journal of Medicine	19	Cape Town, South Africa	H1N1	15 (79%)	Mean (SD): 39.5 (14.8)	6 (32%)	-	-
Nicolay[105]	2010	Critical Care and Resuscitation	76	Ireland	H1N1	37 (49%)	Median (IQR): 43 (30-56)	6 (8%)	-	5 (7%)
Nin[106]	2011	Intensive Care Medicine	84	Argentina, Chile, Uruguay	H1N1	9 (11%)	Mean (SD): AKI: 46 (13), No AKI: 42 (16)	-	-	-
Pettilä[107]	2011	Intensive Care Medicine	628	Australia and New Zealand	H1N1	331 (53%)	Median (IQR): AKI: 47 (34-56), No AKI: 44 (32-55)	116/615 (19%)	-	18 (3%)
Samra[108]	2011	Indian Journal of Anaesthesia	61	New Delhi, India	H1N1	30 (49%)	Mean (SD): 34.2 (12.9)	-	-	-
Tang[109]	2020	Chest	75	Wuhan, China	H1N1	15 (20%)	Median (IQR): 52 (41-64)	15 (20%)	32 (43%)	6 (8%)

Publication	Year	Journal	Study Size (n)	Location	Virus	Female (n, %)	Age	Diabetes Mellitus (n, %)	Hypertension (n, %)	Chronic Kidney Disease (n, %)
Tignanelli[110]	2018	American Journal of Critical Care	57	Michigan, USA	H1N1	21 (36%)	Mean (SD): 41.6 (12.43)	-	-	-
Trimarchi[111]	2010	Journal of Nephrology	22	Buenos Aires, Argentina	H1N1	11 (50%)	Mean (SD): 52.91 (18.89)	2 (9%)	-	4 (18%)
Vallejos[112]	2013	Saudi Journal of Kidney Diseases and Transplantation	44	Buenos Aires, Argentina	H1N1	19 (43%)	34% (18-39 years old), 52% (42-59 years old), 14% (≥60 years old)	3 (7%)	14 (32%)	6 (14%)
Venkata[113]	2010	Mayo Clinic Proceedings	66	Rochester, Minnesota, USA	H1N1	33 (50%)	Mean (SD): 46.9 (17.8)	21 (32%)	31 (47%)	3 (5%)
Yu[114]	2013	Clinical Infectious Diseases	12	Zhejiang Province, China	H7N9	4 (33%)	Median (range): Deceased: 69 (62-86), Survived: 57.5 (37-65)	3 (25%)	7 (58%)	-

c) Non-ACE2-associated viruses

Publication	Year	Journal	Study Size (n)	Location	Virus	Female (n, %)	Age	Diabetes Mellitus (n, %)	Hypertension (n, %)	Chronic Kidney Disease (n, %)
Arabi[115]	2014	Annals of Internal Medicine	12	Saudi Arabia	MERS-CoV	4 (33%)	Median (range): 59 (36-83)	8 (67%)	6 (50%)	5 (42%)
Arabi[116]	2017	Critical Care Medicine	330	Saudi Arabia	MERS-CoV	105 (32%)	Median (IQR): 58 (44-69)	162 (49%)	-	100 (30%)
Dovč[117]	2017	Clinical Nephrology	28	Ljubljana, Slovenia	Seasonal influenza A	11 (39%)	Mean (SD): 57.5 (20.2)	-	-	2 (7%)

Supplemental Table 2. Acute kidney injury (AKI) definitions.

a) COVID-19

Publication	AKI definition
Aggarwal A[27]	KDIGO
Aggarwal S[28]	Increase in sCr > 0.3 mg/dL or > 30% increase in sCr
Al Sulaiman[29]	Not specified
Arentz[30]	KDIGO
Argenziano[31]	Not specified
Azoulay[33]	KDIGO
Bhatraju[34]	KDIGO
Bowe[35]	KDIGO
Burke[36]	Requiring RRT
Cao[37]	Not specified
Chaibi[38]	KDIGO
Chan[39]	KDIGO
Chand[40]	KDIGO
Charytan[41]	AKIN
Chaudri[42]	KDIGO
Cobb[43]	KDIGO
Costa[44]	KDIGO
Cummings[45]	KDIGO
Doher[46]	KDIGO
Dudoignon[47]	KDIGO
Ferguson[48]	KDIGO
Filardo[49]	KDIGO
Fisher[50]	KDIGO
Flythe[51]	Not specified
Fominskiy[52]	KDIGO
Gasparini[53]	KDIGO
Ghosn[54]	KDIGO
Gupta[55]	KDIGO

Publication	AKI definition
Hamilton[56]	KDIGO
Hansrivijit[57]	KDIGO
Hong[58]	KDIGO
Huang C[1]	KDIGO
Isted[59]	KDIGO
Joseph[60]	KDIGO
Kolhe[61]	KDIGO
Larsson[62]	KDIGO
Lee[63]	KDIGO
Li Q[64]	KDIGO
Lowe[65]	KDIGO
Martínez-Rueda[66]	KDIGO
Matthias[67]	KDIGO
Mohamed[69]	KDIGO
Moledina[70]	KDIGO
Mukherjee[71]	KDIGO
Naar[72]	KDIGO
Ng[74]	KDIGO
Okoh[75]	KDIGO
Paek[76]	KDIGO
Qian[77]	KDIGO
Rubin[78]	KDIGO
Samuel[79]	KDIGO
Sang[80]	KDIGO
Suleyman[81]	KDIGO
Taher[82]	KDIGO
Wang D[2]	KDIGO
Wang J[83]	KDIGO
Wang L[84]	KDIGO
Wang P[85]	Not specified
Wang Y[86]	KDIGO

Publication	AKI definition
Wilbers[87]	KDIGO
Xia[88]	KDIGO
Xu Jingyuan[89]	KDIGO
Xu Jiqian[90]	KDIGO
Yan[91]	KDIGO
Yang X[3]	KDIGO
Yu[92]	KDIGO
Zamoner[93]	KDIGO
Zheng[94]	KDIGO

b) ACE2-associated viruses

Publication	AKI definition
Fowler[95]	Urine output <500 mL/day, or creatine level >3.4 mg/dL [>299 μmol/L]
Gomersall[96]	Requiring RRT
Lew[97]	Requiring RRT
Abdulkader[98]	"I" in RIFLE
Bautista[99]	AKIN
Casas-Aparicios[100]	KDIGO
Chaari[101]	RIFLE
Chacko[102]	RIFLE
Demirjian[103]	AKIN
Koegelenberg[104]	AKIN
Nicolay[105]	Requiring dialysis
Nin[106]	"RIF" in RIFLE
Pettïla[107]	"RIF" in RIFLE
Samra[108]	Not specified
Tang[109]	Not specified
Tignanelli[110]	KDIGO

Publication	AKI definition
Trimarchi[111]	RIFLE
Vallejos[112]	Requiring RRT
Venkata[113]	RIFLE
Yu[114]	Not specified

c) Non-ACE2-associated viruses

Publication	AKI definition
Arabi[115]	Requiring RRT
Arabi[116]	Requiring RRT
Dovč[117]	RIFLE

Abbreviations: AKI = acute kidney injury, KDIGO = Kidney Disease Improving Global Outcomes, sCr = serum creatinine, RRT = renal replacement therapy, AKIN = Acute Kidney Injury Network, RIFLE = Risk, Injury, Failure, Loss, End Stage Renal Disease

Supplemental Table 3. Results of meta-regression analysis.

AKI

	Studies with shock data (m= 30)				Studies with vasopressors data (m= 45)			
	Unadjusted		Adjusted		Unadjusted		Adjusted	
**Patient group & comparisons**	**OR (95% CI)**	**P**	**OR (95% CI)**	**P**	**OR (95% CI)**	**P**	**OR (95% CI)**	**P**
Critically ill patients								
ACE2-associated vs. COVID-19	0.77 (0.25, 2.30)	0.634	1.36 (0.53, 3.50)	0.521	2.30 (0.70, 7.61)	0.172	2.62 (0.85, 8.07)	0.093
Non-ACE2-associated vs. COVID-19	1.52 (0.26, 8.91)	0.640	0.96 (0.22, 4.13)	0.958	1.69 (0.34, 8.40)	0.520	1.20 (0.27, 5.44)	0.812
Non-ACE2-associated vs. ACE2-associated	1.99 (0.28, 14.05)	0.489	0.71 (0.13, 3.77)	0.683	0.74 (0.11, 4.93)	0.751	0.46 (0.07, 2.81)	0.399
Prevalence of shock (per 10% increase)			1.31 (1.14, 1.49)	<0.001				
Prevalence of vasopressors use (per 10% increase)							1.26 (1.07, 1.48)	0.005

RRT

	Studies with shock data (m= 25)				Studies with vasopressors data (m= 43)			
	Unadjusted		Adjusted		Unadjusted		Adjusted	
**Patient group & comparisons**	**OR (95% CI)**	**P**	**OR (95% CI)**	**P**	**OR (95% CI)**	**P**	**OR (95% CI)**	**P**
Critically ill patients								
ACE2-associated vs. COVID-19	0.65 (0.20, 2.19)	0.491	1.13 (0.44, 2.91)	0.801	1.63 (0.71, 3.74)	0.251	2.01 (0.96, 4.20)	0.064
Non-ACE2-associated vs. COVID-19	5.49 (1.01, 29.97)	0.049	2.84 (0.77, 10.43)	0.116	4.10 (1.15, 14.60)	0.029	2.92 (0.95, 8.99)	0.062
Non-ACE2-associated vs. ACE2-associated	8.40 (1.20, 58.58)	0.032	2.51 (0.53, 11.87)	0.245	2.52 (0.60, 10.54)	0.206	1.45 (0.40, 5.27)	0.568
Prevalence of shock (per 10% increase)			1.36 (1.18, 1.56)	<0.001				
Prevalence of vasopressors use (per 10% increase)							1.26 (1.11, 1.42)	<0.001

Supplemental Table 4. Risk of bias assessment of included studies

a) COVID-19

Publication	Was the study's target population a close representation of the hospitalized COVID-19 population in relation to relevant variables?	Was the sampling frame a true or close representation of the target population (hospitalized COVID-19 patients)?	Was some form of random selection used to select the sample, or was a census (complete sampling) undertaken?	Is there likely to be minimal bias from incomplete ascertainment of acute kidney injury/renal replacement therapy status in the study population?	Were data collected directly from the subjects?	Was an acceptable definition for acute kidney injury/renal replacement therapy used in the study?	Was the study instrument that defined acute kidney injury/renal replacement therapy shown to have reliability and validity?	Was the same mode of data collection used for all subjects?	Was the length of follow-up to define case definition (acute kidney injury/renal replacement therapy) appropriate?	Were the numerators and denominators for the parameter of interest appropriate?
Aggarwal A	Y	Y	N	N	Y	Y	Y	Y	Y	Y
Aggarwal S	Y	Y	N	N	Y	Y	Y	Y	Y	Y
Al Sulaiman	Y	Y	N	N	Y	N	N	Y	Y	Y
Arentz	Y	Y	N	Y	Y	Y	Y	Y	Y	Y
Argenziano	Y	Y	N	N	Y	N	N	Y	Y	Y
Auld	Y	Y	N	Y	Y	Y	Y	Y	Y	Y
Azoulay	Y	Y	N	Y	Y	Y	Y	Y	Y	Y
Bhatraju	Y	Y	N	Y	Y	Y	Y	Y	Y	Y

Bowe	Y	Y	N	Y	Y	Y	Y	Y	Y	Y
Burke	Y	Y	N	N	Y	N	N	Y	Y	Y
Cao	Y	Y	N	Y	Y	Y	Y	Y	Y	Y
Chaibi	Y	Y	N	Y	Y	Y	Y	Y	Y	Y
Chan	Y	Y	N	Y	Y	Y	Y	Y	Y	Y
Chand	Y	Y	N	Y	Y	Y	Y	Y	V	Y
Charytan	Y	Y	N	Y	Y	Y	Y	Y	Y	Y
Chaudri	Y	Y	N	Y	Y	Y	Y	Y	Y	Y
Cobb	Y	Y	N	Y	Y	Y	Y	Y	Y	Y
Costa	Y	Y	N	Y	Y	Y	Y	Y	Y	Y
Cummings	Y	Y	N	Y	Y	Y	Y	Y	Y	Y
Doher	Y	Y	N	Y	Y	Y	Y	Y	Y	Y
Dudoignon	Y	Y	N	Y	Y	Y	Y	Y	Y	Y
Ferguson	Y	Y	N	Y	Y	Y	Y	Y	Y	Y
Filardo	Y	Y	N	Y	Y	Y	Y	Y	Y	Y
Fisher	Y	Y	N	Y	Y	Y	Y	Y	Y	Y
Flythe	Y	Y	N	N	Y	N	N	Y	Y	Y
Fominskiy	Y	Y	N	Y	Y	Y	Y	Y	Y	Y
Gasparini	Y	Y	N	Y	Y	Y	Y	Y	Y	Y
Ghosn	Y	Y	N	Y	Y	Y	Y	Y	Y	Y
Gupta	Y	Y	N	Y	Y	Y	Y	Y	Y	Y
Hamilton	Y	Y	N	Y	Y	Y	Y	Y	Y	Y

Hansrijivit	Y	Y	N	Y	Y	Y	Y	Y	Y	Y
Hong	Y	Y	N	Y	Y	Y	Y	Y	Y	Y
Huang C	Y	Y	N	Y	Y	Y	Y	Y	Y	Y
Isted	Y	Y	N	Y	Y	Y	Y	Y	Y	Y
Joseph	Y	Y	N	Y	Y	Y	Y	Y	Y	Y
Kolhe	Y	Y	N	Y	Y	Y	Y	Y	Y	Y
Larsson	Y	Y	N	Y	Y	Y	Y	Y	Y	Y
Lee	Y	Y	N	Y	Y	Y	Y	Y	Y	Y
Li Q	Y	Y	N	Y	Y	Y	Y	Y	Y	Y
Lowe	Y	Y	N	Y	Y	Y	Y	Y	Y	Y
Martinez-Rueda	Y	Y	N	Y	Y	Y	Y	Y	Y	Y
Matthias	Y	Y	N	Y	Y	Y	Y	Y	Y	Y
Mitra	Y	Y	N	Y	Y	Y	Y	Y	Y	Y
Mohamed	Y	Y	N	Y	Y	Y	Y	Y	Y	Y
Moledina	Y	Y	N	Y	Y	Y	Y	Y	Y	Y
Mukherjee	Y	Y	N	Y	Y	Y	Y	Y	Y	Y
Naar	Y	Y	N	Y	Y	Y	Y	Y	Y	Y
Ñamendys-Silva	Y	Y	N	Y	Y	Y	Y	Y	Y	Y
Ng	Y	Y	N	Y	Y	Y	Y	Y	Y	Y
Okoh	Y	Y	N	Y	Y	Y	Y	Y	Y	Y
Paek	Y	Y	N	Y	Y	Y	Y	Y	Y	Y
Qian	Y	Y	N	Y	Y	Y	Y	Y	Y	Y

Rubin	Y	Y	N	Y	Y	Y	Y	Y	Y	Y
Samuel	Y	Y	N	Y	Y	Y	Y	Y	Y	Y
Sang	Y	Y	N	Y	Y	Y	Y	Y	Y	Y
Suleyman	Y	Y	N	Y	Y	Y	Y	Y	Y	Y
Taher	Y	Y	N	Y	Y	Y	Y	Y	Y	Y
Wang D	Y	Y	N	Y	Y	Y	Y	Y	Y	Y
Wang J	Y	Y	N	Y	Y	Y	Y	Y	Y	Y
Wang L	Y	Y	N	Y	Y	Y	Y	Y	Y	Y
Wang P	Y	Y	N	N	Y	N	N	Y	Y	Y
Wang Y	Y	Y	N	Y	Y	Y	Y	Y	Y	Y
Wilbers	Y	Y	N	Y	Y	Y	Y	Y	Y	Y
Xia	Y	Y	N	Y	Y	Y	Y	Y	Y	Y
Xu Jingyuan	Y	Y	N	Y	Y	Y	Y	Y	Y	Y
Xu Jigian	Y	Y	N	Y	Y	Y	Y	Y	Y	Y
Yan	Y	Y	N	Y	Y	Y	Y	Y	Y	Y
Yang X	Y	Y	N	Y	Y	Y	Y	Y	Y	Y
Yu	Y	Y	N	Y	Y	Y	Y	Y	Y	Y
Zamoner	Y	Y	N	Y	Y	Y	Y	Y	Y	Y
Zheng	Y	Y	N	Y	Y	Y	Y	Y	Y	Y

b) ACE2-associated viruses

Publication	Was the study's target population a close representation of the hospitalized population in relation to relevant variables?	Was the sampling frame a true or close representation of the target population (hospitalized patients with respiratory infections)?	Was some form of random selection used to select the sample, or was a census (complete sampling) undertaken?	Is there likely to be minimal bias from incomplete ascertainment of acute kidney injury/renal replacement therapy status in the study population?	Were data collected directly from the subjects?	Was an acceptable definition for acute kidney injury/renal replacement therapy used in the study?	Was the study instrument that defined acute kidney injury/renal replacement therapy shown to have reliability and validity?	Was the same mode of data collection used for all subjects?	Was the length of follow-up to define case definition (acute kidney injury/renal replacement therapy) appropriate?	Were the numerators and denominators for the parameter of interest appropriate?
Fowler	Y	Y	N	N	Y	Y	Y	Y	Y	Y
Gomersall	Y	Y	N	N	Y	N	N	Y	Y	Y
Lew	Y	Y	N	N	Y	N	N	Y	Y	Y
Abdulkader	Y	Y	N	Y	Y	Y	Y	Y	Y	Y
Bautista	Y	Y	N	Y	Y	Y	Y	Y	Y	Y
Casas-Aparicio	Y	Y	N	Y	Y	Y	Y	Y	Y	Y
Chaari	Y	Y	N	Y	Y	Y	Y	Y	Y	Y
Chacko	Y	Y	N	Y	Y	Y	Y	Y	Y	Y
Demirjian	Y	Y	N	Y	Y	Y	Y	Y	Y	Y

Koegelenberg	Y	Y	N	Y	Y	Y	Y	Y	Y	Y
Nicolay	Y	Y	N	N	Y	N	N	Y	Y	Y
Nin	Y	Y	N	Y	Y	Y	Y	Y	Y	Y
Pettïla	Y	Y	N	Y	Y	Y	Y	Y	Y	Y
Samra	Y	Y	N	N	Y	N	N	Y	Y	Y
Tang	Y	Y	N	N	Y	N	N	Y	Y	Y
Tignanelli	Y	Y	N	Y	Y	Y	Y	Y	Y	Y
Trimarchi	Y	Y	N	Y	Y	Y	Y	Y	Y	Y
Vallejos	Y	Y	N	Y	Y	Y	Y	Y	Y	Y
Venkata	Y	Y	N	Y	Y	Y	Y	Y	Y	Y
Yu	Y	Y	N	Y	Y	Y	Y	Y	Y	Y

c) Non-ACE2-associated viruses

Publication	Was the study's target population a close representation of the hospitalized population in relation to relevant variables?	Was the sampling frame a true or close representation of the target population (hospitalized patients with respiratory infections)?	Was some form of random selection used to select the sample, or was a census (complete sampling) undertaken?	Is there likely to be minimal bias from incomplete ascertainment of acute kidney injury/renal replacement therapy status in the study population?	Were data collected directly from the subjects?	Was an acceptable definition for acute kidney injury/renal replacement therapy used in the study?	Was the study instrument that defined acute kidney injury/renal replacement therapy shown to have reliability and validity?	Was the same mode of data collection used for all subjects?	Was the length of follow-up to define case definition (acute kidney injury/renal replacement therapy) appropriate?	Were the numerators and denominators for the parameter of interest appropriate?
Arabi	Y	Y	N	N	Y	N	N	Y	Y	Y
Arabi	Y	Y	N	N	Y	N	N	Y	Y	Y
Dovč	Y	Y	N	Y	Y	Y	Y	Y	Y	Y

Appendix A - Search strategy for COVID-19

Search Engine	Search Strategy
Pubmed	(“COVID" OR "COVID-19" OR "2019-nCov" OR "SARS-CoV-2") AND ("kidney injury" OR "renal" OR "kidney" OR "creatinine" OR "renal replacement" OR "dialysis" OR “clinical characteristics” OR “clinical features”)
EMBASE	1 kidney injury.mp. or kidney injury/<br>2 renal failure.mp. or kidney failure/<br>3 acute kidney injury.mp. or acute kidney failure/<br>4 renal replacement therapy.mp. or renal replacement therapy/<br>5 peritoneal dialysis/ or dialysis/ or dialysis.mp.<br>6 hemodialysis.mp. or hemodialysis/<br>7 continuous renal replacement therapy/ or continuous renal replacement.mp.<br>8 1 or 2 or 3 or 4 or 5 or 6 or 7<br>9 COVID-19.mp.<br>10 Coronavirus Disease 2019.mp.<br>11 2019-nCoV.mp.<br>12. SARS-CoV-2.mp.<br>13. 9 or 10 or 11 or 12<br>14. 8 or 13

Restrictions: None.

Appendix B - Search strategy for other ACE2-associated and non-ACE2-associated respiratory viruses

Search Engine	Search Strategy
Pubmed	("flu" OR "influenza" OR "influenza H1N1" OR "influenza H7N9" OR "influenza H5N1" OR "influenza H3N2" OR "SARS" OR "severe acute respiratory syndrome" OR "SARS-CoV-1" OR "MERS" or "middle east respiratory syndrome" or "MERS-CoV") AND ("kidney injury" OR "renal" OR “renal failure” OR "kidney" OR "creatinine" OR "renal replacement" OR "dialysis" OR "clinical characteristics" OR "clinical features")
EMBASE	1 kidney injury.mp. or kidney injury/<br>2 renal failure.mp. or kidney failure/<br>3 acute kidney injury.mp. or acute kidney failure/<br>4 renal replacement therapy.mp. or renal replacement therapy/<br>5 peritoneal dialysis/ or dialysis/ or dialysis.mp.<br>6 hemodialysis.mp. or hemodialysis/<br>7 continuous renal replacement therapy/ or continuous renal replacement.mp.<br>8 1 or 2 or 3 or 4 or 5 or 6 or 7<br>9 Influenza A virus/ or influenza B/ or avian influenza/ or "Influenza A virus (H5N3)"/ or "influenza A (H7N7)"/ or Influenza B virus/ or Asian influenza/ or "Influenza A virus (H5N8)"/ or 2009 H1N1 influenza/ or "Influenza A virus (H2N2)"/ or "Influenza A virus (H5N2)"/ or influenza.mp. or pandemic influenza/ or "influenza A (H3N2)"/ or seasonal influenza/ or "influenza A (H3N8)"/ or "avian influenza (H5N1)"/ or influenza A/ or "Influenza A virus (H1N1)"/ or "influenza A (H5N1)"/ or "Influenza A virus (H5N1)"/ or "Influenza A virus (H3N2)"/ or "Influenza A virus (H9N2)"/ or influenza/ or "influenza A (H2N2)"/ or avian influenza virus/ or "influenza A (H1N1)"/ or "Influenza A virus (H7N9)"/ or "influenza A (H7N9)"/<br>10 severe acute respiratory syndrome.mp. or severe acute respiratory syndrome/<br>11 SARS.mp. or severe acute respiratory syndrome/<br>12 MERS.mp.<br>13 middle east respiratory syndrome.mp. or Middle East respiratory syndrome/<br>14 flu.mp. or influenza/<br>15 1 or 2 or 3 or 4 or 5 or 6 or 7<br>16 8 or 9 or 10 or 11 or 12 or 13<br>17 15 and 16

Restrictions: None
